# A receptor-like kinase recognizes viral proteins at the *trans*-Golgi network/early endosome and inhibits infection in rice

**DOI:** 10.1038/s41421-025-00847-4

**Published:** 2025-12-16

**Authors:** Huacai Wang, Yawen Liu, Mengting Zhang, Rongxiang Fang, Yongsheng Yan

**Affiliations:** 1 Institute of Microbiology, Chinese Academy of Sciences, Beijing, China; 2 Innovation Academy for Seed Design, Chinese Academy of Sciences, Beijing, China; 3 CAS Center for Excellence in Biotic Interactions, University of Chinese Academy of Sciences, Beijing, China

**Keywords:** Plant sciences, Molecular biology

## Abstract

Receptor-like kinases (RLKs) reside on the cell surface and recognize apoplastic colonization by plant-infecting microbes to initiate immune responses. Whether RLKs can also recognize intracellular colonization by viruses to activate antiviral defense mechanisms in plants remains unknown. Here, we report the identification and characterization of a *trans*-Golgi network/early endosome (TGN/EE)-localized RLK that recognizes viral proteins and inhibits infection in rice. OsVIRK1, a cysteine-rich receptor-like kinase, promotes rice resistance to rice stripe virus (RSV), one of the most devastating viruses of rice. *OsVIRK1* transcription is induced in RSV-infected rice, and its protein accumulates through autophosphorylation and redox-mediated regulation. OsVIRK1 physically interacts with the RSV coat protein (CP), a known immune elicitor, and nonstructural protein 3 (NS3), an antiviral RNA-silencing suppressor, at the TGN/EE. OsVIRK1 is required for CP-triggered defense gene expression. It phosphorylates NS3, reducing NS3 accumulation in the cytoplasm and thus repressing its activity as an RNA-silencing suppressor. Our findings suggest that OsVIRK1 recognizes viral proteins at the TGN/EE to inhibit infection by activating plant antiviral immunity and dampening viral counterdefense.

## Introduction

Plants are constantly challenged by diverse pathogens, including viruses, bacteria, fungi, and oomycetes. To perceive and ward off potential pathogens, plants have developed a highly resilient, two-tiered innate immune detection-and-response system: pattern recognition receptor (PRR)-mediated pattern-triggered immunity (PTI) and intracellular nucleotide-binding domain leucine-rich repeat receptor (NLR)-mediated effector-triggered immunity (ETI)^1–3^. In turn, host-adapted pathogens typically evade or counteract the plant immune system by impairing host recognition or subsequent immune responses through a variety of mechanisms^4,5^.

Receptor-like kinases (RLKs) are known to function as PRRs, which are typically located at the cell surface and sense apoplastic pathogens such as bacteria and fungi by detecting their pathogen-associated molecular patterns (PAMPs). They then initiate PTI responses such as calcium influx, reactive oxygen species (ROS) bursts, and activation of mitogen-activated protein kinase (MAPK) cascades^6–8^. The molecular mechanisms of RLK-mediated PTI signaling — from ligand recognition to PRR complex formation and immune response activation — have been well characterized through studies of well-known PRRs such as FLS2, EFR, and LYK5 in *Arabidopsis*, in which co-receptor recruitment and receptor-like cytoplasmic kinase (RLCK) activation via phosphorylation constitute the core system^6–8^.

Whether plant RLKs can recognize viral infection is unknown, although increasing evidence suggests that uncharacterized cell surface-localized PRR(s) that recognize viruses are likely to exist^9,10^. For example, viral nucleic acids and coat protein (CP) have been proposed to function as PAMPs to induce typical PTI responses^11,12^. The PRR co-receptor BAK1 is required for plant defense against several RNA viruses^13^, and a number of viral proteins have been shown to inhibit the ROS burst and MAPK cascade activation^14–16^. In particular, as viruses are obligate intracellular pathogens, it would be very interesting to know whether plant hosts perceive virus-derived immune signals intracellularly and activate antiviral defenses, as has been documented in mammals^17,18^. The rice RBR-type E3 ligase RBRL was recently shown to act as a nucleus-localized sensor, perceiving viral CPs to activate jasmonate signaling and downstream antiviral pathways^19^. Here, we report that the RLK OsVIRK1 is localized at the *trans*-Golgi network/early endosome (TGN/EE) and recognizes viral proteins to inhibit viral infection in rice.

## Results

### *OsVIRK1* plays a positive role in rice resistance to RSV

**Fig. 1:**
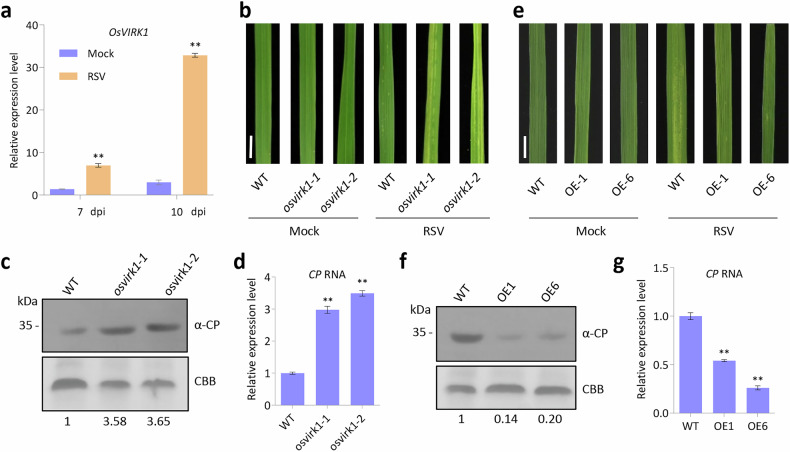
Virus-induced *OsVIRK1* positively regulates rice anti-RSV defense. **a** RSV infection induces *OsVIRK1* expression. Rice seedlings grown for 10 days were inoculated with RSV or mock control and collected at 7 and 10 days post inoculation (dpi) for total RNA extraction and RT-qPCR analysis. **b** *osvirk1* mutants showing reduced resistance to RSV after 10 dpi. **c** Western blot showing that *osvirk1* mutants accumulated more RSV CP protein than the WT at 10 dpi. **d** RT-qPCR analysis showing that *osvirk1* mutants accumulated more RSV CP RNA than the WT at 10 dpi. **e** *OsVIRK1-FLAG-HA*-overexpressing lines (OE1 and OE6) showing enhanced resistance to RSV at 15 dpi. **f** Western blot showing that OE1 and OE6 accumulated less RSV CP protein than the WT at 15 dpi. **g** RT-qPCR analysis showing that OE1 and OE6 accumulated less RSV CP RNA than the WT at 15 dpi. In **a,** **d**, **g**, values are means ± SD of three biological replicates; **P < 0.01, Student’s *t*-test. In **b**, **e**, scale bars, 1 cm. In **c**, **f**, normalized intensities of CP signals are indicated below the blot panels; Rubisco large subunit bands were visualized by Coomassie Brilliant Blue (CBB) staining and served as a loading control. All experiments were repeated at least twice with similar results.

Rice stripe virus (RSV) belongs to the genus *Tenuivirus* and is a filamentous, negative-strand RNA virus that often causes severe damage to rice growth and production in most Asian countries^20^. To explore the functions and regulatory mechanisms of RLKs in plant defense against RSV infection, we generated CRISPR/Cas9-derived mutant materials for several rice *RLK* genes induced by RSV infection^21,22^ and then subjected them to RSV infection assays. Two mutants (*osvirk1-1* and *osvirk1-2*) with frameshift mutations in *virus-inducible receptor-like kinase 1* (*OsVIRK1*, *LOC_Os05g41370*), which is highly induced by RSV infection, were highly susceptible to RSV infection. They showed more severe disease symptoms and greater accumulation of viral CP protein and RNA than wild-type (WT) plants after 10 days of RSV infection (Fig. 1a–d; Supplementary Fig. S1a), indicating that knockout of *OsVIRK1* reduced rice defenses against RSV infection. By contrast, the CRISPR/Cas9-derived mutant of another RSV-induced rice RLK gene (*LOC_Os01g05980*) showed no obvious alteration in CP accumulation upon RSV infection (Supplementary Fig. S1b, c). To further examine the function of OsVIRK1 in the rice response to RSV infection, we transformed the 35S promoter-driven *OsVIRK1* coding sequence with a 3′-terminal *FLAG-HA* tag fusion into rice to produce 35S::*OsVIRK1-FLAG-HA* transgenic rice plants. The *OsVIRK1*-overexpressing transgenic rice lines (OsVIRK1-FLAG-HA-OE lines) were confirmed by western blotting and RT-qPCR (Supplementary Fig. S1d, e) and then used for an RSV infection assay. In contrast to the *osvirk1* mutants, the OsVIRK1-FLAG-HA-OE rice plants displayed enhanced resistance to RSV, with weaker disease symptoms and reduced accumulation of CP protein and RNA than the WT after 15 days of RSV infection (Fig. 1e–g), indicating that overexpression of *OsVIRK1* enhanced rice resistance to RSV infection. Taken together, these results suggest that the virus-inducible gene *OsVIRK1* plays a positive role in rice defense against RSV.

### OsVIRK1 localizes to the plasma membrane and TGN/EE

**Fig. 2:**
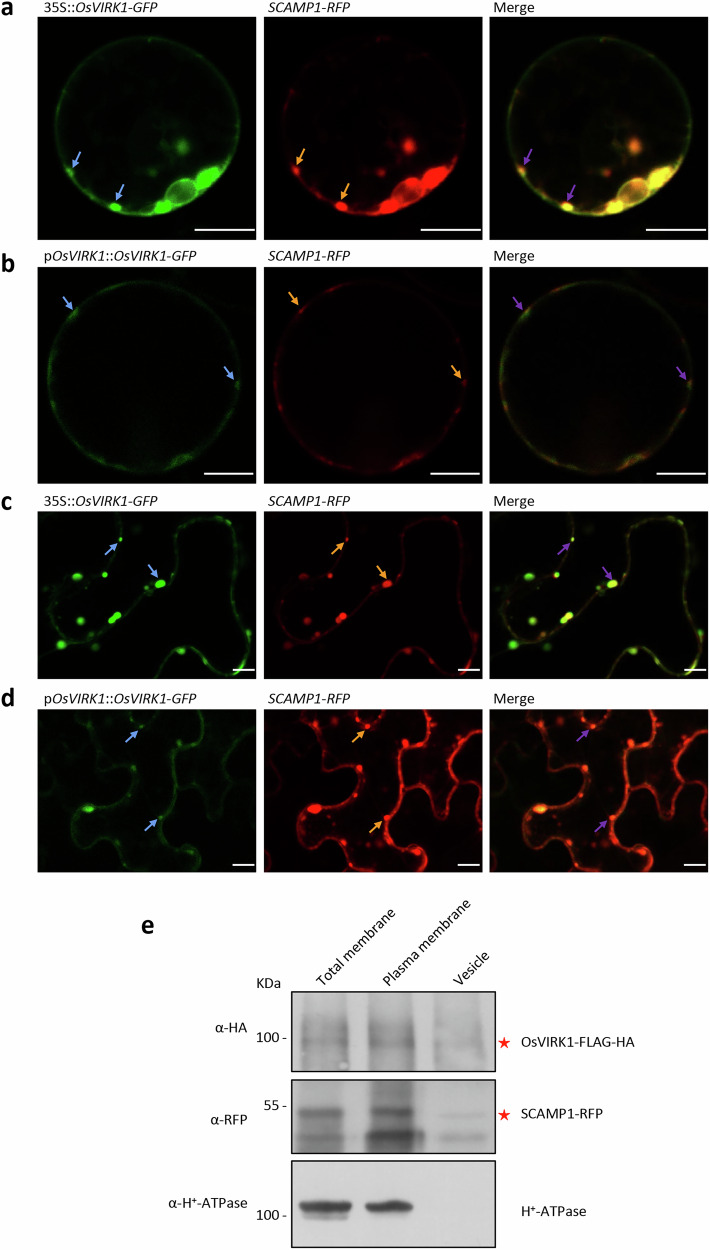
OsVIRK1 co-localizes with the TGN/EE marker SCAMP1. **a**, **c** 35S promoter-driven *OsVIRK1-GFP* was transiently co-expressed with *SCAMP1-RFP* in rice protoplasts (**a**) and *N*. *benthamiana* leaves (**c**). **b**, **d** *OsVIRK1* native promoter-driven *OsVIRK1-GFP* was transiently co-expressed with *SCAMP1-RFP* in rice protoplasts (**b**) and *N*. *benthamiana* leaves (**d**). The GFP and RFP fluorescence signals were observed by confocal laser microscopy. Arrows indicate representative fluorescence signals of puncta. Scale bars, 10 μm. **e** Membrane fractionation assay showing the colocalization of OsVIRK1-FLAG-HA and SCAMP1-RFP in the plasma membrane and vesicle fractions. These fractions were prepared from protoplasts of *OsVIRK1-FLAG-HA-*overexpressing rice seedlings transiently expressing *SCAMP1-RFP*. Western blot was performed with anti-HA, anti-RFP, or anti-H^+^-ATPase antibody. All experiments were repeated two or three times with similar results.

OsVIRK1 encodes a cysteine-rich receptor-like kinase (CRK) of 680 amino acids (aa) that contains a signal peptide (aa 1–36), an extracellular receptor region with two conserved DUF26 domains containing four Cys residues (aa 91–147 and aa 208–260, respectively), a single-pass transmembrane region (aa 297–319), and an intracellular Ser/Thr protein kinase region (aa 348–651) (http://rice.plantbiology.msu.edu/; Supplementary Fig. S2a). RLKs typically localize to the plasma membrane (PM) via the endoplasmic reticulum (ER)-Golgi apparatus-TGN-PM secretory pathway^23–26^. To characterize the subcellular localization of OsVIRK1, we transiently expressed a *GFP*-fused version of *OsVIRK1* under the control of the 35S promoter (35S::*OsVIRK1-GFP*) in rice protoplasts and *N. benthamiana* leaves, and then observed the resulting GFP fluorescence by confocal microscopy. Consistent with its expected localization at the PM, OsVIRK1-GFP was localized at the periphery of epidermal pavement cells and protoplast cells (Fig. 2a, c; Supplementary Fig. S2b, c). OsVIRK1-GFP signals were also detected at intracellular punctate structures (Fig. 2a, c; Supplementary Fig. S2b, c). Close observation revealed that the puncta were membrane-enveloped bodies and aggregates of membrane-enveloped bodies, and OsVIRK1-GFP was located at the membranes of these bodies (Supplementary Fig. S2d). By contrast, OsCRK10, a homolog of OsVIRK1, was predominantly localized at the PM of rice protoplasts and *N. benthamiana* leaf cells (Supplementary Fig. S2e, f), similar to previously reported CRKs, e.g., AtCRK2/4/6/36^27,28^. When *OsVIRK1-GFP* was transiently expressed in rice protoplasts and *N. benthamiana* leaves under the control of the native *OsVIRK1* promoter (p*OsVIRK1*::*OsVIRK1-GFP*), it showed the same subcellular localization patterns as 35S::*OsVIRK1-GFP*, although p*OsVIRK1*::*OsVIRK1-GFP* produced weaker GFP fluorescence signals (Fig. 2b, d).

To further confirm that OsVIRK1 is localized to the PM, we stained OsVIRK1-GFP-expressing rice protoplast cells with the amphiphilic styryl dye FM4-64. OsVIRK1-GFP colocalized with the PM-associated FM4-64 dye, confirming the localization of OsVIRK1-GFP at the PM (Supplementary Fig. S3a). To determine whether OsVIRK1-GFP-labeled puncta originated from the secretory Golgi apparatus and the TGN, we co-expressed OsVIRK1-GFP with the Golgi marker (MANI-RFP^29^) or the TGN marker (SCAMP1-RFP^29^) in *N. benthamiana* leaves. The OsVIRK1-GFP and SCAMP1-RFP puncta colocalized completely (Fig. 2a–d; Supplementary Fig. S2d). The OsVIRK1-GFP puncta did not colocalize with typical Golgi bodies labeled by MANI-RFP (Supplementary Fig. S3b), but they appeared to partially colocalize with MANI-RFP-positive aggregates (Supplementary Fig. S3b), which may represent Golgi-associated TGN^30,31^ and have been used as a TGN marker^32^. PM-resident RLK proteins, such as the brassinosteroid receptor BRI1 and the immune receptor FLS2, are often internalized into the vacuole for degradation via the PM-early endosome (EE)-late endosome (LE)-vacuole endocytic pathway^23–26^. To investigate whether the OsVIRK1-associated puncta were endocytic vesicles, we co-expressed OsVIRK1-GFP with VPS23A-RFP or ARA7-RFP, two markers of endocytic vesicles, in *N. benthamiana* leaves. The punctate signals of OsVIRK1-GFP did not colocalize with VPS23A-RFP (an endosomal sorting complex required for transport (ESCRT)-I component^32^) or ARA7-RFP (a late endosomal marker^32^) in *N. benthamiana* leaves (Supplementary Fig. S3c, d). PM proteins can be degraded by autophagy^23,25^. To test whether OsVIRK1-GFP-marked puncta were associated with the autophagosome, OsVIRK1-GFP was expressed with the autophagy marker ATG8-RFP in *N. benthamiana* leaves. No colocalization of their puncta was observed (Supplementary Fig. S3e). As SCAMP1 is also an EE marker and the TGN also serves as an EE^29^, the above observations together suggest that OsVIRK1 is mainly located on the PM and TGN/EE, and this result was confirmed by a membrane fractionation assay using OsVIRK1-FLAG-HA-overexpressing rice plants (Fig. 2e).

### Autophosphorylation of OsVIRK1 maintains its protein accumulation

**Fig. 3:**
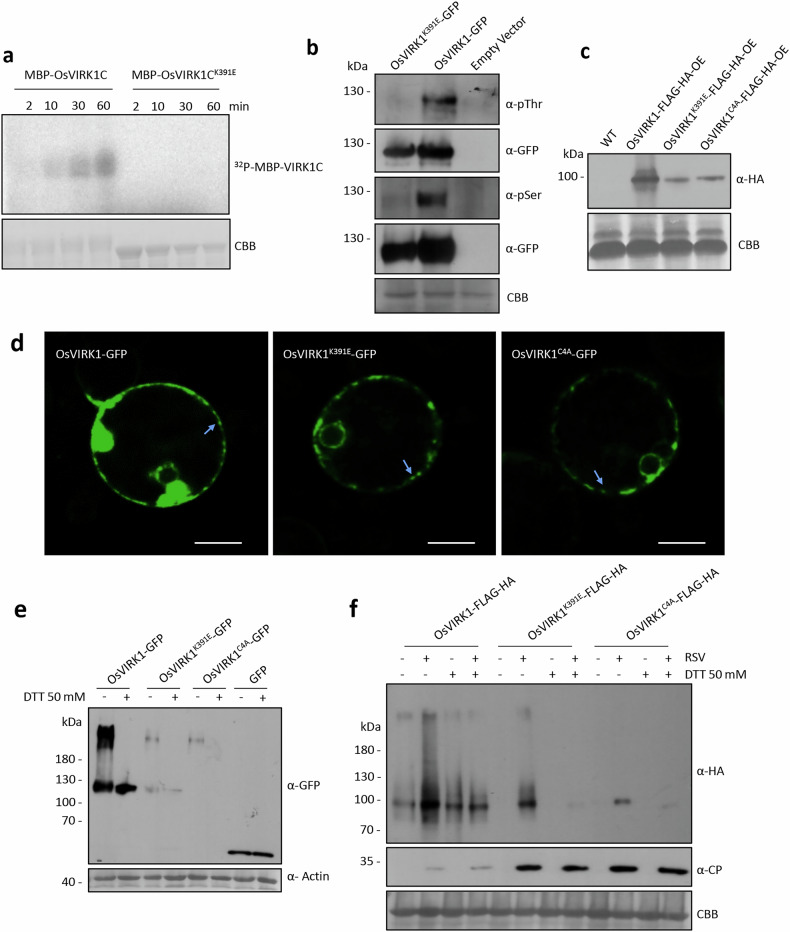
Autophosphorylation and redox regulation promote the accumulation of OsVIRK1. **a** Autophosphorylation analysis of OsVIRK1 in vitro. The MBP-fused C-terminal region of OsVIRK1 (MBP-OsVIRK1C) and 391 Lys-mutated OsVIRK1C (MBP-OsVIRK1C^K391E^) were purified and subjected to an in vitro kinase activity assay. **b** Autophosphorylation analysis of OsVIRK1 in vivo. OsVIRK1-GFP, OsVIRK1^K391E^-GFP, and the control vector were transiently expressed in rice protoplasts. Total proteins were extracted for anti-GFP immunoprecipitation and subsequent anti-pThr and anti-pSer immunoblotting. **c** Anti-HA western blot assay showing the accumulation of OsVIRK1-FLAG-HA, OsVIRK1^K391E^-FLAG-HA, and OsVIRK1^C4A^-FLAG-HA proteins in the corresponding transgenic rice lines. **d** Subcellular localization of OsVIRK1-GFP, OsVIRK1^K391E^-GFP, and OsVIRK1^C4A^-GFP proteins in rice protoplasts. Arrows indicate representative fluorescence signals of puncta. Scale bars, 10 μm. **e** Analysis of the redox regulation of OsVIRK1 in *N*. *benthamiana*. OsVIRK1-GFP, OsVIRK1^K391E^-GFP, or OsVIRK1^C4A^-GFP was transiently expressed in *N*. *benthamiana* leaves. **f** Analysis of the redox regulation of OsVIRK1 in rice. Transgenic rice constitutively expressing OsVIRK1-FLAG-HA, OsVIRK1^K391E^-FLAG-HA, or OsVIRK1^C4A^-FLAG-HA was inoculated with RSV or mock. In **e**, **f**, total protein was extracted with or without DTT (50 mM) and separated by non-reducing SDS-PAGE, followed by western blot analysis with anti-GFP, anti-HA, or anti-CP antibodies. In **a**–**c**, **f**, the Rubisco large subunit bands were visualized by Coomassie Brilliant Blue (CBB) staining and served as a loading control. In **e**, actin was used as a loading control. All experiments were repeated two or three times with similar results.

To assess whether OsVIRK1 is an active kinase, we expressed and purified the cytoplasmic kinase region of OsVIRK1 fused to the MBP tag (MBP-VIRK1C) from *Escherichia coli*. As a negative control, the highly conserved Lys residue (K391) required for CRK kinase activity in the ATP-binding domain of OsVIRK1 was mutated to Glu residue (E391) to produce the MBP-VIRK1C^K391E^ protein. The two fusion proteins were subjected to an in vitro kinase activity assay. The autophosphorylation signals of MBP-VIRK1C were clearly detected, but those of kinase-dead MBP-VIRK1C^K391E^ were not (Fig. 3a), indicating that OsVIRK1 is an active kinase that can undergo autophosphorylation in vitro. The phosphorylation of OsVIRK1-GFP (immunoprecipitated (IP) by anti-GFP beads), but not λPP-treated OsVIRK1-GFP or OsVIRK1^K391E^-GFP, was detected with anti-pThr and anti-pSer antibodies when transiently expressed in rice protoplasts (Fig. 3b) and *N*. *benthamiana* leaves (Supplementary Fig. S4a, b), suggesting that OsVIRK1 can also undergo autophosphorylation in vivo. Indeed, phosphorylation of VIRK1-FLAG-HA was detected in OsVIRK1-FLAG-HA-OE rice plants, with or without RSV infection (Supplementary Fig. S4c). The autophosphorylated MBP-VIRK1C was subjected to mass spectrometry analysis, and six amino acid residues, T360, S364, T396, T451, S492, and T528, were readily identified as potential autophosphorylation sites (Supplementary Table S1). To examine whether these six potential autophosphorylation sites contribute to the autophosphorylation of OsVIRK1-GFP in vivo, we constructed OsVIRK1^6A^-GFP, in which all six autophosphorylation sites were mutated to Ala. OsVIRK1-GFP and OsVIRK1^6A^-GFP were transiently expressed in *N. benthamiana* leaves, and their phosphorylation levels were detected. Compared to OsVIRK1-GFP, OsVIRK1^6A^-GFP showed almost no phosphorylation, even when protein accumulation was increased by co-expressing the RSV suppressor nonstructural protein 3 (NS3) (Supplementary Fig. S4d), suggesting that the six amino acid residues are key autophosphorylation sites of OsVIRK1.

We noticed that the accumulation of OsVIRK1^K391E^-GFP and OsVIRK1^6A^-GFP was frequently lower than that of OsVIRK1-GFP in the anti-GFP IP assays (Fig. 3b; Supplementary Fig. S4a, b, d), suggesting that the loss of autophosphorylation reduces OsVIRK1 protein accumulation. Indeed, when transiently expressed in *N. benthamiana* leaves, the abundance of OsVIRK1^K391E^-GFP and OsVIRK1^6A^-GFP was significantly lower than that of OsVIRK1-GFP (Supplementary Fig. S5a, b). Similarly, the accumulation of OsVIRK1^K391E^-FLAG-HA in 35S::*OsVIRK1*^*K391E*^*-FLAG-HA* transgenic rice plants was significantly lower than that of OsVIRK1-FLAG-HA in 35S::*OsVIRK1-FLAG-HA* transgenic rice plants (Fig. 3c). Treatment of 35S::*OsVIRK1*^*K391E*^*-FLAG-HA* transgenic rice plants with the protein synthesis inhibitor cycloheximide (CHX) revealed that OsVIRK1^K391E^ was prone to degradation (Supplementary Fig. S5c, d). Treatment with the proteasome inhibitor MG132 or the protease inhibitor E64d partially rescued the accumulation of OsVIRK1^K391E^-FLAG-HA in 35S::*OsVIRK1*^*K391E*^*-FLAG-HA* transgenic rice plants (Supplementary Fig. S5c, d). These observations indicate that autophosphorylation is required to maintain OsVIRK1 protein levels.

To test whether the autophosphorylation of OsVIRK1 affects its subcellular localization, kinase-dead OsVIRK1^K391E^-GFP was transiently expressed in rice protoplasts and *N. benthamiana* leaves. OsVIRK1^K391E^-GFP exhibited similar localization patterns to OsVIRK1-GFP, although with reduced GFP fluorescence intensity and fewer puncta (Fig. 3d; Supplementary Fig. S6a). Colocalization analysis confirmed that the punctate signals of OsVIRK1^K391E^-GFP colocalized with the TGN/EE marker SCAMP1-RFP (Supplementary Fig. S6b). Like OsVIRK1^K391E^-GFP, OsVIRK1^6A^-GFP showed similar subcellular localization patterns to OsVIRK1-GFP, with reduced GFP fluorescence intensity and fewer puncta (Supplementary Fig. S6c). These observations suggest that loss of autophosphorylation does not affect the subcellular localization of OsVIRK1 despite reducing OsVIRK1 protein accumulation.

### Redox regulation of OsVIRK1 controls its protein accumulation

Like most plant CRKs, OsVIRK1 possesses two copies of the DUF26 domain (Supplementary Fig. S2a). The four Cys residues in each DUF26 domain are potentially capable of forming intramolecular and intermolecular Cys bridges, which are redox-sensitive and important for protein folding, structure, and stability^33–35^. We therefore investigated whether OsVIRK1 undergoes redox regulation involving the Cys residues of the DUF26 domains. To this end, we transiently expressed OsVIRK1-MYC and OsVIRK1-GFP in *N. benthamiana* leaves, then extracted total proteins with or without the addition of the reducing agent dithiothreitol (DTT) and subjected them to non-reducing SDS-PAGE followed by immunoblot analysis. In addition to the low-molecular-weight (MW) proteins, high-MW complexes of OsVIRK1-GFP and OsVIRK1-MYC were also detected in the absence of DTT (Fig. 3e; Supplementary Fig. S7a, b). The addition of DTT to the extracts almost completely eliminated the high-MW complexes (Fig. 3e; Supplementary Fig. S7a, b), suggesting that OsVIRK1 undergoes redox regulation in planta.

To determine whether the Cys residues of the two OsVIRK1 DUF26 domains confer the redox regulation of OsVIRK1, the four conserved Cys residues (C110, C138, C223, C251) of the two DUF26 domains were simultaneously mutated to Ala (A110, A138, A223, A251) to generate Cys-mutated OsVIRK1-GFP (OsVIRK1^C4A^-GFP). OsVIRK1^C4A^-GFP was transiently expressed in *N. benthamiana* leaves, and its protein accumulation was analyzed by non-reducing SDS-PAGE. The results revealed little accumulation of low- or high-MW Cys-mutated OsVIRK1-GFP proteins (Fig. 3e), indicating that the mutated Cys residues mediate the redox regulation of OsVIRK1 and are critical for its accumulation. Consistent with these results, reducing SDS-PAGE showed that the accumulation of OsVIRK1^C4A^-GFP was reduced compared to that of OsVIRK1-GFP when transiently expressed in *N. benthamiana* leaves (Supplementary Fig. S5a). Likewise, the accumulation of OsVIRK1^C4A^-FLAG-HA in 35S::*OsVIRK1*^*C4A*^*-FLAG-HA* transgenic rice plants was lower than that of OsVIRK1-FLAG-HA in 35S::*OsVIRK1-FLAG-HA* transgenic rice plants (Fig. 3c). Treatment of 35S::*OsVIRK1*^*C4A*^*-FLAG-HA* transgenic rice plants with the protein synthesis inhibitor CHX revealed that OsVIRK1^C4A^ was prone to degradation (Supplementary Fig. S5c, d). However, treatment with the proteasome inhibitor MG132 or the protease inhibitor E64d did not increase the accumulation of OsVIRK1^C4A^-FLAG-HA in 35S::*OsVIRK1*^*C4A*^*-FLAG-HA* transgenic rice plants (Supplementary Fig. S5c, d). Like OsVIRK1^K391E^-GFP, OsVIRK1^C4A^-GFP showed similar localization patterns to OsVIRK1-GFP, as shown by its complete colocalization with the TGN/EE marker SCAMP1 (Supplementary Fig. S6b), although the GFP fluorescence intensity and number of puncta were reduced (Fig. 3d; Supplementary Fig. S6a), indicating that mutation of these Cys residues did not affect the subcellular localization of OsVIRK1.

Rice infected by RSV has been reported to accumulate ROS and regulate redox status to defend against viral infection^36^. To confirm that OsVIRK1 is subjected to redox regulation to control its accumulation during the response of rice to RSV infection, 35S::*OsVIRK1-FLAG-HA* and 35S::*OsVIRK1*^*C4A*^*-FLAG-HA* transgenic rice plants were used to observe the protein conformations of OsVIRK1-FLAG-HA and OsVIRK1^C4A^-FLAG-HA, with and without RSV infection, using non-reducing SDS-PAGE. Regardless of RSV infection, OsVIRK1-FLAG-HA formed high-MW complexes, which were reduced to low-MW complexes by the addition of DTT in 35S::*OsVIRK1-FLAG-HA* transgenic rice plants (Fig. 3f). In addition, RSV infection increased the accumulation of both low- and high-MW complexes of OsVIRK1-FLAG-HA in 35S::*OsVIRK1-FLAG-HA* transgenic rice plants (Fig. 3f). By contrast, only low-MW complexes of OsVIRK1^C4A^-FLAG-HA were detected in RSV-infected 35S::*OsVIRK1*^*C4A*^*-FLAG-HA* transgenic rice plants (Fig. 3f). Together, these results suggest that OsVIRK1 undergoes redox regulation through the Cys residues of its two DUF26 domains, which in turn regulates its protein accumulation in rice in response to RSV.

### OsVIRK1 that lacks the kinase active site and conserved Cys residues cannot protect rice against viral infection

35S::*OsVIRK1-FLAG-HA*, 35S::*OsVIRK1*^*K391E*^*-FLAG-HA*, and 35S::*OsVIRK1*^*C4A*^*-FLAG-HA* transgenic rice plants were next subjected to RSV infection assays. In contrast to the increased RSV resistance of OsVIRK1-FLAG-HA-OE plants, OsVIRK1^K391E^-FLAG-HA-OE and OsVIRK1^C4A^-FLAG-HA-OE transgenic plants showed RSV infection comparable to that of WT plants, as shown by measurement of RSV CP levels (Fig. 3f; Supplementary Fig. S7c). Therefore, OsVIRK1 that lacks the kinase active site or the conserved Cys residues cannot protect rice against viral infection. Consistent with this finding, OsVIRK1^6A^-GFP phosphorylation was almost completely abolished, and protein accumulation was reduced (Supplementary Figs. S4d and S5b), and rice plants overexpressing OsVIRK1^6A^-GFP did not show increased resistance to RSV infection (Supplementary Fig. S7d).

### OsVIRK1 interacts with RSV CP at the TGN/EE

**Fig. 4:**
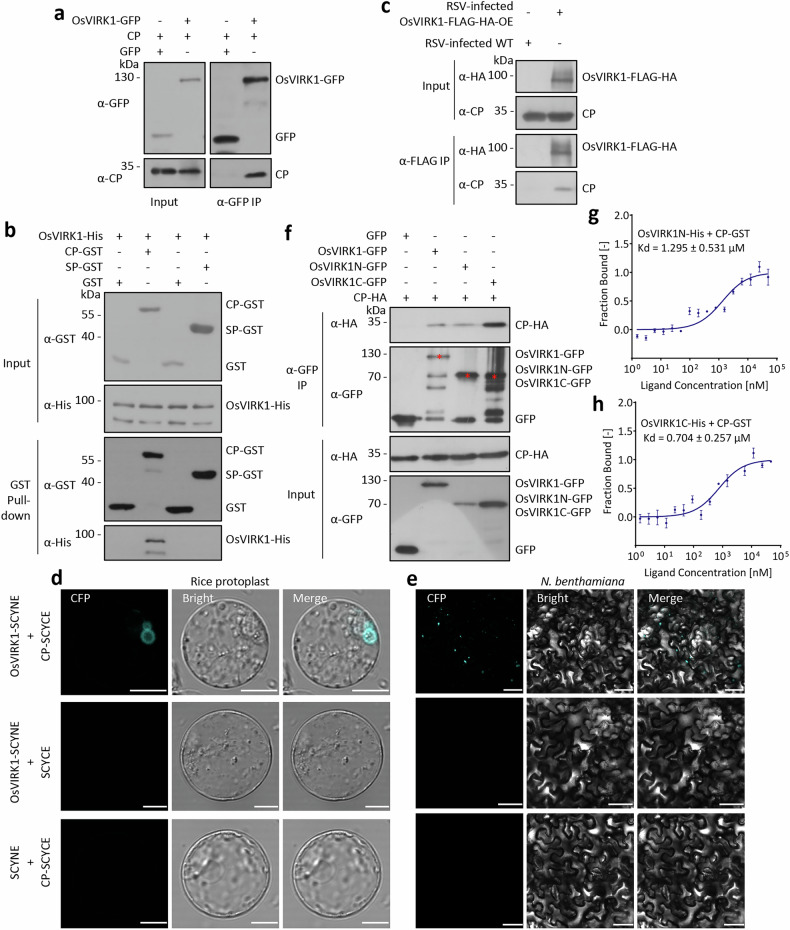
OsVIRK1 physically interacts with RSV CP. **a** Co-IP assay showing that OsVIRK1 is associated with CP in *N*. *benthamiana*. OsVIRK1-GFP and CP or SP were transiently co-expressed in *N*. *benthamiana* leaves. The proteins were subjected to anti-GFP IP followed by western blot analysis with anti-GFP, anti-CP, and anti-SP antibodies. **b** GST pull-down assay showing that OsVIRK1 binds to CP, but not SP, in vitro. His-tagged OsVIRK1 (OsVIRK1-His), GST-tagged CP (CP-GST) and SP (SP-GST), and GST recombinant proteins were affinity-purified, and protein–protein interactions were examined by GST pull-down assay. **c** Co-IP assay showing that OsVIRK1 is associated with CP in RSV-infected rice. Total proteins were extracted from RSV-infected WT and OsVIRK1-FLAG-HA-OE rice plants, followed by anti-FLAG co-IP and subsequent western blot analysis with anti-HA and anti-CP antibodies. **d**, **e** BiFC assay showing that OsVIRK1 interacts with CP at the TGN/EE. The designated construct pairs were co-transfected into rice protoplasts and *N*. *benthamiana* leaves, and the CFP fluorescence signals were observed. Scale bars, 10 μm (**d**) or 50 μm (**e**). **f** Co-IP assay showing that both the N- and C-terminal regions of OsVIRK1 are associated with CP in *N*. *benthamiana*. GFP, OsVIRK1-GFP, OsVIRK1N-GFP, or OsVIRK1C-GFP was transiently co-expressed with CP-HA in *N*. *benthamiana* leaves. The proteins were then subjected to anti-GFP IP and subsequent western blot analysis using anti-GFP and anti-HA antibodies. The red stars mark the expected protein bands in the western blots. **g**, **h** MST analysis showing the binding affinity between OsVIRK1N-His or OsVIRK1C-His and CP-GST. All experiments were repeated two or three times with similar results.

GFP-tagged RSV CP localizes to the PM and forms numerous puncta in the cytoplasm, similar to OsVIRK1-GFP when transiently expressed in *N. benthamiana* leaves^37^. In addition, CP-YFP and SCAMP1-RFP puncta partially colocalized when co-expressed in *N. benthamiana* leaves (Supplementary Fig. S8a). We therefore tested whether CP was associated with OsVIRK1 in planta. To this end, we co-expressed CP and OsVIRK1-GFP or GFP in *N. benthamiana* leaves, and then performed α-GFP co-immunoprecipitation (co-IP). CP was co-immunoprecipitated with OsVIRK1-GFP, but not with GFP alone (Fig. 4a). In addition, α-HA co-IP showed that OsVIRK1-FLAG-HA, but not the two PM-localized proteins AtLYK4-FLAG-HA and AtLYK5-FLAG-HA, interacted with CP when they were co-expressed in *N. benthamiana* leaves (Supplementary Fig. S8b). Further, GST pull-down assay using purified proteins from *E. coli* (Supplementary Fig. S9a) showed that the CP-GST fusion protein, but not the GST control, bound to OsVIRK1-His in vitro (Fig. 4b). By contrast, RSV SP and SP-GST showed no interaction with OsVIRK1-GFP or OsVIRK1-His in co-IP and GST pull-down assays, respectively (Fig. 4b; Supplementary Fig. S8c). To confirm that OsVIRK1 interacts with CP in RSV-infected rice, we performed an α-FLAG co-IP assay with RSV-infected WT and OsVIRK1-FLAG-HA-OE rice plants. OsVIRK1-FLAG-HA was co-immunoprecipitated with CP in RSV-infected OsVIRK1-FLAG-HA-OE rice plants, but not in RSV-infected WT plants (Fig. 4c). To determine whether OsVIRK1 interacts with CP at the PM or TGN/EE, we performed bimolecular fluorescence complementation (BiFC) assays by co-transfecting OsVIRK1-fused SCYNE (the N-terminal fragment of CFP) and CP-fused SCYCE (the C-terminal fragment of CFP) into rice protoplasts and *N. benthamiana* leaves. The BiFC results indicated that OsVIRK1 interacted with CP at the TGN/EE in plant cells, as shown by the observation that CFP fluorescence signals exhibited punctate or ring-like structures and colocalized with SCAMP1-RFP (Fig. 4d, e; Supplementary Fig. S8d, e). Together, these results provide evidence that OsVIRK1 physically interacts with CP at the TGN/EE in RSV-infected rice. In addition, the closest homolog of OsVIRK1, OsCRK46 (LOC_Os10g17960), could interact with CP in a co-IP assay (Supplementary Fig. S8f) and may therefore function redundantly.

To evaluate which region of OsVIRK1 binds to RSV CP, the N-terminal (aa 1–263) and C-terminal (aa 319–680) regions of OsVIRK1 were separately fused with GFP and co-expressed with CP-HA in *N. benthamiana* leaves for α-GFP co-IP assays. Both the N-terminal region (OsVIRK1N-GFP) and the C-terminal region (OsVIRK1C-GFP) of OsVIRK1 interacted with CP-HA (Fig. 4f; Supplementary Fig. S8g). Consistent with this finding, His pull-down assays showed direct binding of CP-GST to both OsVIRK1N-His and OsVIRK1C-His in vitro (Supplementary Fig. S8h, i). In addition, a microscale thermophoresis (MST) analysis revealed that CP-GST, but not the GST control, interacted with OsVIRK1N-His and OsVIRK1C-His with low equilibrium dissociation constants (*K*_d_) (Fig. 4g, h; Supplementary Fig. S9b–d).

The N-terminal region (aa 1–47) of RSV CP is required for its self-interaction and oligomerization^37,38^. It has also been suggested that RSV CP forms a groove structure required for RNA binding that is dependent on the amino acids Arg^77^, Thr^79^, Asp^80^, Lys^87^, and Arg^122^. CP variants with mutations in these amino acids, CP^R77A/T79A/D80A/K87A^ and CP^R122A^, have been shown to lose the RNA-binding ability^38^. To test whether the self-interaction or RNA-binding ability of CP affects its interaction with OsVIRK1, CP^∆1-47^-HA (with the N-terminal 47 amino acids deleted) and CP^R77A/T79A/D80A/K87A^-HA and CP^R122A^-HA (with the indicated amino acid mutations) were separately co-expressed with OsVIRK1-GFP in *N. benthamiana* leaves for co-IP assays. CP^∆1-47^-HA showed almost no accumulation (Supplementary Fig. S8g), indicating that the CP self-interaction region is critical for CP accumulation. By contrast, CP^R77A/T79A/D80A/K87A^-HA and CP^R122A^-HA were co-immunoprecipitated with OsVIRK1-GFP, like WT CP-HA (Supplementary Fig. S8g), indicating that the loss of RNA-binding ability does not affect the interaction of CP with OsVIRK1.

### OsVIRK1 is required for CP-triggered defense gene expression

**Fig. 5:**
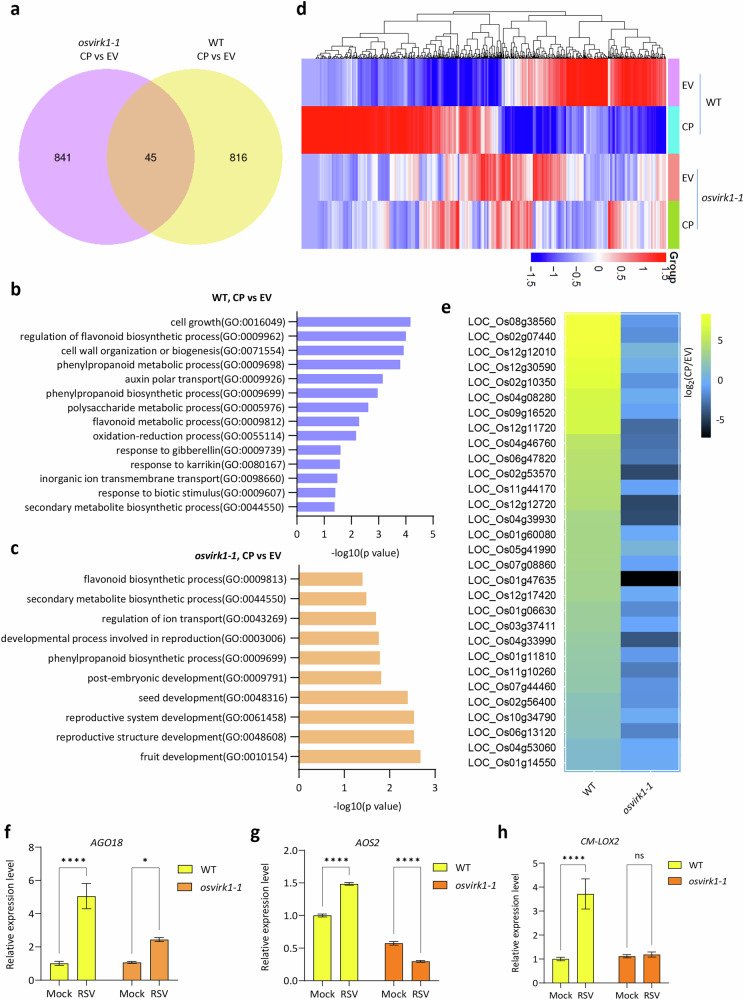
OsVIRK1 is required for CP-triggered defense gene expression. **a** Numbers of DEGs in CP-treated WT and *osvirk1-1* rice protoplasts shown as a Venn diagram. **b** The most enriched GO terms in the DEGs from CP-treated WT. **c** The most enriched GO terms in the DEGs from CP-treated *osvirk1-1*. **d** Heatmap of up- and downregulated DEGs from CP-treated WT and *osvirk1-1* rice protoplasts. **e** Heatmap of expression fold changes of defense-related DEGs from CP-treated WT and *osvirk1-1* rice protoplasts. In **a**–**d**, CP or the empty vector (EV) was transfected into WT and *osvirk1-1* rice protoplasts, and whole-genome mRNA transcriptome analysis was performed to compare gene expression and function. **f**–**h** RT-qPCR analysis of the expression of three genes upregulated by RSV infection in the WT and os*virk1-1* mutant inoculated for 20 days with Mock or RSV. Values are means ± SD of three biological replicates. *P < 0.05, ****P < 0.0001, ns not significant, two-way ANOVA.

It has been suggested that RSV CP is an elicitor of immune responses, as ectopic expression of CP increases rice resistance to RSV^39–42^. It has also been proposed that CP is recognized by an unknown receptor that activates antiviral defense in rice^42^. Since OsVIRK1 interacts with CP at the TGN/EE and is highly induced to defend against RSV infection (Figs. 1, 2), we wondered whether OsVIRK1 perceives CP to activate antiviral immunity. To test this hypothesis, we investigated whether OsVIRK1 is required for CP-triggered expression of antiviral defense genes. To this end, we transiently expressed CP in WT and *osvirk1* rice protoplasts (Supplementary Fig. S10a, b) and performed whole-genome mRNA transcriptome analysis. Differentially expressed genes (DEGs) were identified using the criteria (log_2_(fold change) ≥ 1 and *P* < 0.05). Compared with WT expressing the empty vector (EV), WT expressing CP contained 861 DEGs: 472 upregulated and 389 downregulated (Fig. 5a; Supplementary Table S2). Compared with *osvirk1* expressing the EV, *osvirk1* expressing CP contained 361 upregulated DEGs and 525 downregulated DEGs, only 45 of which were shared with those in CP-treated WT (Fig. 5a; Supplementary Table S2). Gene Ontology (GO) analysis showed that the 861 DEGs in CP-treated WT were mainly assigned to biological terms (−log_10_(*P* value) > 2), potentially associated with the regulation of plant growth, metabolism, and defense. These included “cell growth,” “cell wall organization or biogenesis,” “phenylpropanoid metabolic process,” “auxin polar transport,” “phenylpropanoid biosynthetic process,” “polysaccharide metabolic process,” “flavonoid metabolic process,” and “oxidation-reduction process” (Fig. 5b). By contrast, the 886 DEGs in CP-treated *osvirk1* were mainly assigned to biological terms related to plant development (−log_10_(*P* value) > 2), such as “fruit development,” “reproductive structure development,” “reproductive system development,” and “seed development” (Fig. 5c). Most of the genes that were upregulated in CP-treated WT were not upregulated in CP-treated *osvirk1* (Fig. 5d). In particular, CP-treated WT showed significant upregulation of 30 defense genes, including genes encoding the disease resistance protein RGA2, MLO-like protein OsMLO8, calmodulin-binding protein 60b isoform, NB-LRR protein Xa38, defensin-like DEFL protein DEFL78, and LRR receptor-like kinase, none of which were upregulated in CP-treated *osvirk1* (Fig. 5e; Supplementary Table S2). Together, these observations suggest that OsVIRK1 is required for CP-activated defense gene expression in rice protoplasts. Furthermore, the induced expression levels of *AGO18*, *AOS2*, and *CM-LOX2*^42^ were lower in RSV-infected *osvirk1* than in RSV-infected WT (Fig. 5f–h), suggesting that OsVIRK1 is necessary for the virus-induced expression of some defense genes.

### OsVIRK1 interacts with RSV NS3 at the TGN/EE

**Fig. 6:**
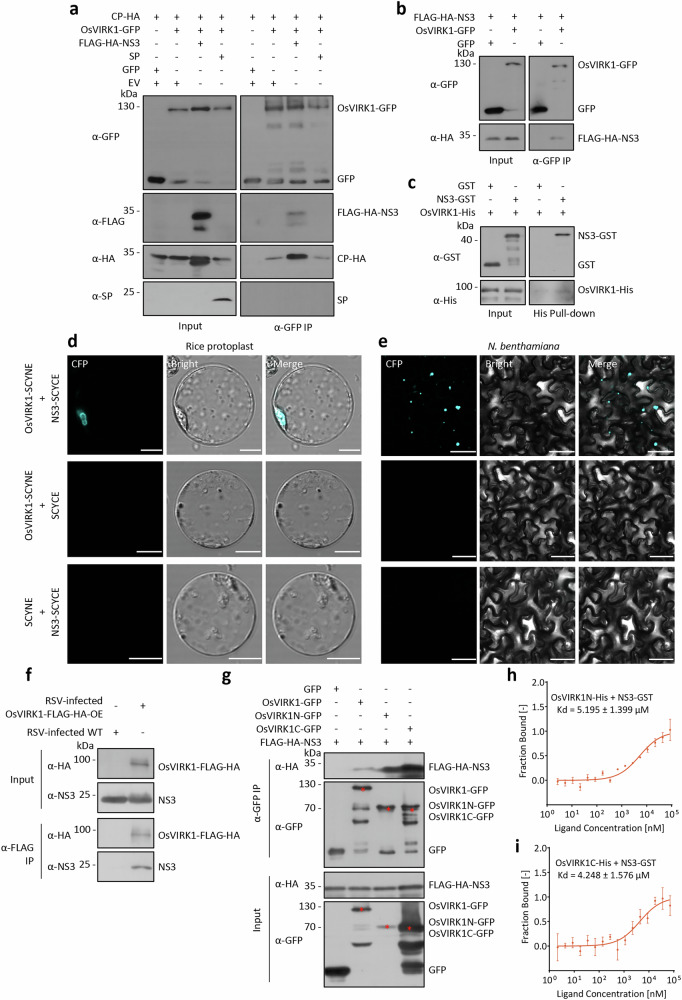
OsVIRK1 physically interacts with RSV NS3. **a** Co-IP assay showing that OsVIRK1 is associated with CP and NS3 in *N*. *benthamiana*. OsVIRK1-GFP, CP-HA, and FLAG-HA-NS3 or SP were transiently co-expressed in *N*. *benthamiana* leaves, and the proteins were subjected to anti-GFP IP followed by western blot analysis with anti-GFP, anti-HA, anti-FLAG, and anti-SP antibodies. **b** Co-IP assay showing that OsVIRK1 is associated with NS3 in *N*. *benthamiana*. OsVIRK1-GFP or GFP was transiently co-expressed with FLAG-HA-NS3 in *N*. *benthamiana* leaves, and the proteins were subjected to anti-GFP IP followed by western blot analysis with anti-GFP and anti-HA antibodies. **c** His pull-down assay showing that OsVIRK1 binds to NS3 in vitro. His-tagged OsVIRK1 (VIRK1-His) and GST-tagged NS3 (NS3-GST) recombinant proteins were affinity-purified, and protein–protein interactions were examined by His pull-down assay. **d**, **e** BiFC assay showing that OsVIRK1 interacts with NS3 at the TGN/EE. The designated construct pairs were co-transfected into rice protoplasts and *N*. *benthamiana* leaves, and the CFP fluorescence signals were observed. Scale bars are 10 μm (**d**) or 50 μm (**e**). **f** Co-IP assay showing that OsVIRK1 is associated with NS3 in RSV-infected rice. Total proteins were extracted from RSV-infected WT and OsVIRK1-FLAG-HA-OE rice plants for anti-FLAG co-IP, followed by western blot analysis with anti-HA and anti-NS3 antibodies. **g** Co-IP assay showing that both the N- and C-terminal regions of OsVIRK1 are associated with NS3 in *N*. *benthamiana*. GFP, OsVIRK1-GFP, OsVIRK1N-GFP, or OsVIRK1C-GFP was transiently co-expressed with FLAG-HA-NS3 in *N*. *benthamiana* leaves, and the proteins were subjected to anti-GFP IP followed by western blot analysis with anti-GFP and anti-HA antibodies. The red stars mark the expected protein bands in the western blots. **h** MST analysis showing the binding affinity between OsVIRK1N-His or OsVIRK1C-His and NS3-GST. All experiments were repeated two or three times with similar results.

Yeast two-hybrid and BiFC assays have suggested that CP interacts with SP and NS3, two major pathogenicity determinants of RSV^20,37^. To test whether SP and NS3 affect the interaction between CP and OsVIRK1, SP or FLAG-HA-NS3 was co-expressed with CP-HA and OsVIRK1-GFP in *N. benthamiana* leaves to perform an α-GFP co-IP assay. The addition of FLAG-HA-NS3 or SP did not affect the interaction of CP-HA with OsVIRK1-GFP (Fig. 6a). Interestingly, the α-GFP co-IP assay also showed that, in addition to CP-HA, FLAG-HA-NS3, but not SP, was co-immunoprecipitated with OsVIRK1-GFP (Fig. 6a), suggesting an association of OsVIRK1 with NS3 in planta. We therefore tested whether NS3 interacts directly with OsVIRK1. To this end, we co-expressed FLAG-HA-NS3 with OsVIRK1-GFP or GFP in *N. benthamiana* leaves for an α-GFP co-IP assay and purified NS3-GST and OsVIRK1-His fusion proteins to perform a His pull-down assay. The results of both assays indicated that NS3 interacted with OsVIRK1 in vivo and in vitro (Fig. 6b, c). BiFC assays indicated that OsVIRK1 interacted with NS3 at the TGN/EE in rice protoplasts and *N. benthamiana* leaves, as the CFP fluorescence signals formed by the interaction of OsVIRK1-fused SCYNE with NS3-fused SCYCE exhibited punctate or ring-like structures and colocalized with SCAMP1-RFP (Fig. 6d, e; Supplementary Fig. S11a, b). To confirm that OsVIRK1 interacts with NS3 in RSV-infected rice, we performed an α-FLAG co-IP assay using RSV-infected WT and OsVIRK1-FLAG-HA-OE rice plants. OsVIRK1-FLAG-HA was co-immunoprecipitated with NS3 in RSV-infected OsVIRK1-FLAG-HA-OE rice plants, but not in RSV-infected WT plants (Fig. 6f). Furthermore, co-IP and His pull-down assays showed that NS3 bound to both OsVIRK1N and OsVIRK1C (Fig. 6g; Supplementary Fig. S8h, i). MST analysis demonstrated that NS3-GST interacted with OsVIRK1N-His and OsVIRK1C-His with low *K*_d_ (Fig. 6h, i; Supplementary Fig. S9b–d). Together, these results suggest that OsVIRK1 physically interacts not only with CP but also with NS3 at the TGN/EE in RSV-infected rice.

### OsVIRK1 phosphorylates RSV NS3 to dampen its RNA-silencing suppressor activity

**Fig. 7:**
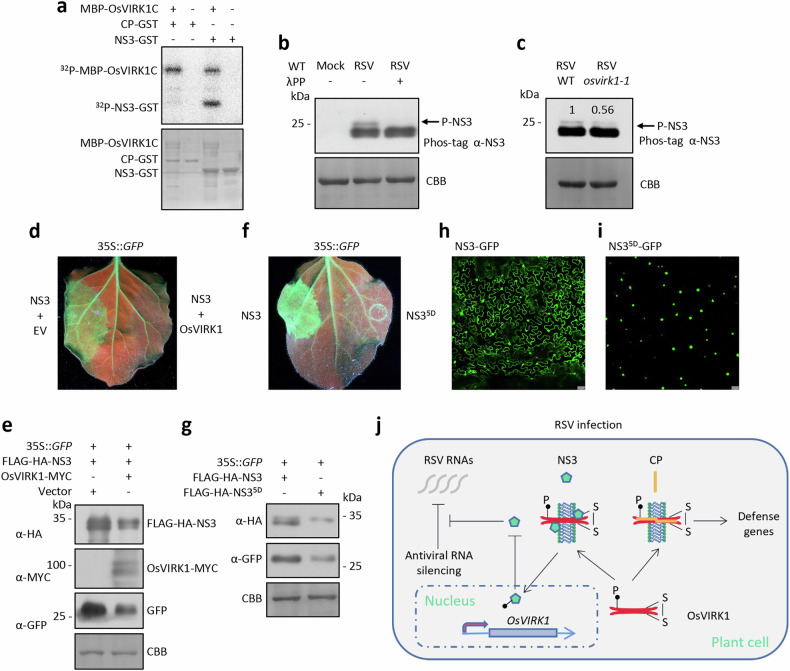
OsVIRK1 phosphorylates RSV NS3 to dampen its VSR function. **a** OsVIRK1C phosphorylates NS3. Purified NS3-GST or CP-GST was incubated with purified MBP-OsVIRK1C and subjected to an in vitro kinase activity assay. **b** Phos-tag SDS-PAGE coupled immunoblotting assay showing that OsVIRK1-phosphorylated NS3 in RSV-infected WT rice, and this phosphorylation was diminished by the addition of λPP. **c** Phos-tag SDS-PAGE coupled immunoblotting assay showing that OsVIRK1 is required for NS3 phosphorylation in RSV-infected rice. The numbers represent the normalized intensities of the phosphorylated NS3 signals. **d** The VSR function of NS3 was drastically reduced when co-expressed with OsVIRK1-MYC. The VSR activity of NS3 was assessed by transient co-expression of 35S::*GFP* and FLAG-HA-NS3 with or without OsVIRK1-MYC in 16c leaves. GFP fluorescence was imaged 5 days post agroinfiltration. EV, empty vector. **e** OsVIRK1 reduces NS3 accumulation. Western blot analysis of the accumulation of GFP, FLAG-HA-NS3, and OsVIRK1-MYC in the materials described in **d**. **f** The phosphomimic NS3^5D^ lost VSR function as assessed by transient co-expression of 35S::*GFP* with FLAG-HA-NS3 or FLAG-HA-NS3^5D^ in 16c leaves. GFP fluorescence was imaged 5 days post agroinfiltration. **g** Compared to NS3, the phosphomimic NS3^5D^ showed reduced protein accumulation. Western blot analysis of the accumulation of GFP, FLAG-HA-NS3, and FLAG-HA-NS3^5D^ in the materials described in **f**. **h**, **i** Subcellular localization of NS3-GFP and NS3^5D^-GFP proteins in *N. benthamiana* leaves; NS3-GFP and NS3^5D^-GFP were expressed in different zones of one leaf. All experiments were repeated two or three times with similar results. **j** A model illustrating that OsVIRK1 recognizes CP and NS3 and inhibits RSV infection. After infection, RSV produces the viral proteins CP and NS3 and viral RNAs in plant cells. Plant antiviral RNA silencing mediates cleavage of the viral RNAs and inhibits infection, and this is counteracted by the viral suppressor NS3. *OsVIRK1* expression is induced by RSV infection. OsVIRK1 protein accumulates through autophosphorylation and redox regulation and localizes to the TGN/EE, where it recognizes CP and NS3. Recognition of CP by OsVIRK1 activates the expression of antiviral defense genes. OsVIRK1 directly phosphorylates NS3, promoting and reducing NS3 accumulation in the nucleus and cytoplasm, respectively, thereby inhibiting its VSR activity and releasing antiviral RNA silencing.

MBP-VIRK1C phosphorylated NS3-GST, but not CP-GST, in an in vitro kinase activity assay (Fig. 7a). The phosphorylated NS3-GST was subjected to mass spectrometry analysis, and five amino acid residues (S117, T118, T133, S182, and S188) were readily identified as potential sites of NS3 phosphorylation by OsVIRK1 (Supplementary Table S3). In a Phos-tag SDS-PAGE coupled immunoblotting assay, two phosphorylated NS3 bands were detected for the in vitro kinase reaction containing MBP-VIRK1C and NS3-GST (Supplementary Fig. S12a). One of the bands disappeared when the five amino acid residues S117, T118, T133, S182, and S188 were simultaneously mutated to non-phosphorylatable alanine (NS3^5A^) (Supplementary Fig. S12a), indicating that OsVIRK1 phosphorylates these five residues and other unknown amino acid residues in vitro. The five amino acid residues appeared to be the major phosphorylation sites, as the signal intensity of the phosphorylated NS3 band generated from these residues was stronger than that of the other phosphorylated NS3 band generated from unknown amino acid residues in the Phos-tag SDS-PAGE immunoblotting assay (Supplementary Fig. S12a). This assay also indicated that NS3 was phosphorylated in RSV-infected WT rice, and this phosphorylation was reduced in RSV-infected *osvirk1* rice (Fig. 7b, c). Together, these observations suggest that OsVIRK1 phosphorylates NS3 in RSV-infected rice.

NS3 functions as a viral suppressor of RNA silencing (VSR) by binding small RNAs^43,44^. Whether OsVIRK1 affects the VSR activity of NS3 was tested using the *GFP* transgenic 16c *N. benthamiana* system, in which 35S::*GFP* transiently expressed in 16c leaves triggers silencing of *GFP* mRNA. In this system, NS3 interfered with 35S::*GFP-*triggered RNA silencing, resulting in clear GFP fluorescence and protein accumulation in 16c leaves (Fig. 7d, e), as reported previously^43^. Co-expression of OsVIRK1 and NS3 inhibited NS3 accumulation and almost completely blocked NS3-mediated suppression of 35S::*GFP-*triggered RNA silencing, as shown by marked reductions in GFP fluorescence intensity and protein accumulation (Fig. 7d, e). By contrast, AtLYK4 did not affect NS3-mediated suppression of 35S::*GFP-*triggered RNA silencing (Supplementary Fig. S12b). In addition, OsVIRK1 alone or co-expressed with 35S::*GFP* in 16c leaves did not affect GFP accumulation (Supplementary Fig. S12c). These observations indicate that the interaction of OsVIRK1 with NS3 dampens the VSR activity of NS3, potentially by reducing NS3 accumulation. To further clarify whether OsVIRK1 inhibits NS3 protein accumulation and VSR activity by phosphorylating NS3, we mutated the five potentially OsVIRK1-phosphorylated sites of NS3 (S117, T118, T133, S182, and S188) to produce a phosphomimic version (NS3^5D^, all changed to Asp). As expected, NS3^5D^ showed reduced accumulation and lost VSR activity compared to the WT NS3 when expressed in 16c leaves (Fig. 7f, g). Interestingly, subcellular localization analysis showed that NS3-GFP was localized in both the cytoplasm and the nucleus, as reported previously^37,43^ (Fig. 7h). By contrast, NS3^5D^-GFP was localized exclusively to the nucleus (Fig. 7i). Since NS3 functions as an antiviral RNA-silencing suppressor by binding to small RNAs in the cytoplasm^43,44^, its impaired accumulation in the cytoplasm may be the reason for the loss of VSR activity in NS3^5D^. When NS3^5D^-GFP was transiently expressed in *N. benthamiana* leaves treated with the protein degradation inhibitors ED64a and MG132, it was still localized exclusively to the nucleus (Supplementary Fig. S13a), suggesting that the impaired cytoplasmic accumulation of NS3^5D^-GFP was not due to degradation of cytoplasm-localized NS3^5D^-GFP. Together, these results suggest that OsVIRK1 may phosphorylate NS3 to block its cytoplasmic localization, thereby reducing its accumulation in the cytoplasm and dampening its VSR activity.

We next investigated the effect of each of the five amino acid residues on NS3 accumulation, subcellular localization, and VSR activity upon phosphorylation by OsVIRK1. To this end, we separately mutated the five amino acid residues to aspartic acid to generate the phosphomimic versions NS3^S117D^, NS3^T118D^, NS3^T133D^, NS3^S182D^, and NS3^S188D^. Compared to WT NS3, NS3^S117D^ and NS3^S182D^ showed significant reductions in accumulation when transiently expressed in *N. benthamiana* leaves, NS3^T118D^ and NS3^T133D^ showed slight reductions, and NS3^S188D^ showed no reduction (Supplementary Fig. S12d). These results suggest that S117 and S182 have major roles and S118 and T133 have minor roles in the reduced accumulation of NS3 upon phosphorylation by OsVIRK1 (Fig. 7e). We next examined the subcellular localization of NS3^S117D^-GFP, NS3^T118D^-GFP, NS3^T133D^-GFP, NS3^S182D^-GFP, and NS3^S188D^-GFP. Like NS3^5D^-GFP (Fig. 7i), NS3^S182D^-GFP was predominantly localized to the nucleus (Supplementary Fig. S12e), and its cytoplasmic accumulation was not increased by treatment with ED64a or MG132 (Supplementary Fig. S13b), indicating that phosphorylation at S182 blocks the cytoplasmic localization of NS3. In addition, NS3^S117D^ and NS3^S182D^, but not the other three NS3 mutants, exhibited significantly reduced VSR activities compared to that of WT NS3 (Supplementary Fig. S12f–k), consistent with their markedly reduced accumulation (Supplementary Fig. S12d). Together, these observations suggest that S117 and S182 are two main phosphorylation sites that contribute to OsVIRK1-mediated reduction of NS3 cytoplasmic accumulation and VSR activity.

## Discussion

Viruses remodel cytoplasmic membranes and use membrane-associated pathways, such as the ER-Golgi-TGN secretory pathway and the endosomal sorting pathway, to support formation of the virus replication factory, viral assembly, intra- and intercellular movement of viral complexes, and inhibition of host responses^45–48^. In mammals, both PM-resident and endomembrane organelle (e.g., endosome)-resident PRRs have been shown to recognize virus-derived PAMPs (e.g., viral coat proteins and nucleic acids) and initiate pattern-triggered antiviral immunity^17,18^. In plants, the existence of PM- or endomembrane organelle-localized PRRs and the process by which plant hosts recognize viral components to activate defense mechanisms remain to be explored^9,10^. Although RLKs such as NIK1 and BAM1/2 have been shown to play key roles in plant defense against viral infection^49–51^, it remains unknown whether RLKs can directly recognize viral components to activate defense in plants. Viruses are obligate intracellular parasites, and the question of whether RLKs can sense viral immune signals via their intracellular domains has also been raised^10^. In this study, we demonstrated that OsVIRK1 functions as an RLK and recognizes RSV CP and NS3 at the TGN/EE to defend against viral infection by facilitating CP-triggered defense gene expression and phosphorylating NS3 to inhibit its VSR activity (Fig. 7j). RSV is the type member of the genus *Tenuivirus*^52^. It would be interesting to explore whether other species in this genus and their corresponding hosts exhibit similar recognition mechanisms.

It is well known that plant RLKs function as PRRs to recognize extracellular ligands through their ectodomains^7,53^. Both the N- and C-terminal regions of OsVIRK1 can recognize RSV proteins at the TGN/EE (Figs. 4 and 6), suggesting that some plant RLKs can recognize immune signals at the endomembrane as well as the PM. Although both OsVIRK1 and CP/NS3 can localize to the PM and TGN/EE (Fig. 2; Supplementary Figs. S8a and S12e), BiFC assays only detected their interactions at the TGN/EE (Figs. 4d, e and 6d, e). It may be that OsVIRK1 and CP/NS3 are distributed dynamically in specific nanodomains on the PM or that their interactions are too weak to be observed in the BiFC assays. Furthermore, it is unknown how OsVIRK1 binds to the two proteins and whether it forms a complex with them or interacts with them at different microdomains when carrying out its antiviral function. This antiviral function requires both OsVIRK1 kinase activity and protein stability (Fig. 3). It appears that neither of the two main protein degradation machineries — the ubiquitin-proteasome system and autophagy — are responsible for the instability of the OsVIRK1 protein when it lacks the kinase active site and conserved Cys residues (Supplementary Fig. S5c, d), suggesting that other mechanisms regulate its protein stability.

RLKs have been shown to undergo dynamic subcellular trafficking to maintain protein localization and homeostasis. For instance, FLS2 is transported to the PM via the secretory pathway, and it also enters two distinct endosomal pathways: the recycling endosomal pathway, which is responsible for shuttling it between the PM and the TGN/EE, and the late endosomal trafficking pathway, which degrades it upon ligand-dependent activation^23–26^. OsVIRK1 did not show colocalization with the late endosome marker protein ARA7 (Supplementary Fig. S3d), which suggests that it does not enter the late endosomal trafficking pathway. Further study is needed to determine whether OsVIRK1 makes use of the recycling endosomal pathway to maintain protein homeostasis and how it is retained at the TGN/EE.

In plants, RLK-mediated immune recognition at the PM triggers PTI responses, which typically include ROS production, MAPK activation, callose deposition, and so forth. Atypical immune recognition, however, activates non-canonical immune pathways. For example, the rice RBR-type E3 ligase RBRL has recently been shown to act as a nuclear sensor that recognizes viral CPs, activating jasmonate signaling and downstream antiviral pathways^19^. OsVIRK1 activates defense gene expression and phosphorylates a viral RNA-silencing suppressor (Figs. 5 and 7). However, it remains unclear whether TGN/EE-localized OsVIRK1-mediated recognition of CP and NS3 activates typical PTI responses. Furthermore, the mechanism by which OsVIRK1 subsequently activates defense gene expression upon CP perception at the TGN/EE remains to be elucidated. To counter PTI-mediated defense, bacterial and fungal pathogens use effectors to interfere with the activity and functions of PTI pathway components^4,5^. Viral proteins such as geminivirus C4 have also been shown to act as virulence effectors, binding to FLS2 and inhibiting early PTI responses^15^. It will be very interesting to examine whether CP, NS3, and other RSV proteins have a role in interfering with OsVIRK1 activity and function.

It is well known that the release of viral envelope protein into the host cytoplasm and its dissociation from viral nucleic acids are early steps that occur simultaneously during viral infection. In plants, Dicer protein-mediated RNA silencing, which cleaves viral double-stranded RNAs into small RNAs, is thought to be the first layer of antiviral immunity^54,55^. The recognition of CP by OsVIRK1 suggests that RLK-mediated antiviral immunity is also a primary defense mechanism in plants. Antiviral RNA silencing is typically disrupted by virus-encoded RNA-silencing suppressors as a counter-defense mechanism^54,55^. OsVIRK1 directly recognizes and phosphorylates NS3, thereby altering its subcellular distribution in the nucleus and cytoplasm and repressing its VSR activity, suggesting a counter-counter-defense role for OsVIRK1 in reactivating antiviral RNA silencing. Further study is needed to determine the mechanisms by which phosphorylation results in exclusion of NS3 from the cytoplasm and loss of its VSR activity.

OsVIRK1 is a CRK-type RLK. CRKs are among the largest groups of RLKs in plants and play important roles in plant growth and adaptation to biotic and abiotic stresses^56,57^. They have been shown to participate in plant PTI by regulating ROS production, callose deposition, MAPK activation, and pathogen sensitivity^58^. Although no direct immune ligands of CRKs have been identified, increasing evidence suggests that CRKs act as components of PRR complexes. For example, AtCRK28 associates with the FLS2/BAK1 immune complex and is required for cell death, whereas AtCRK2 directly interacts with and phosphorylates RBOHD to regulate ROS production upon pathogen infection^58^. It remains unclear whether PM-localized OsVIRK1 also functions in the regulation of plant immunity. The ectodomains of CRKs contain two DUF26 domains with a conserved cysteine motif, which suggests that CRKs undergo redox regulation and are key components of the ROS sensing machinery or ROS sensors^59^. Indeed, OsVIRK1 undergoes redox regulation to maintain its protein accumulation, which is crucial for its antiviral function (Fig. 3e, f). As RSV infection promotes ROS accumulation^36^, further study is needed to establish whether OsVIRK1 senses and transfers the ROS signal in order to defend against viral infection.

## Materials and methods

### Plasmid construction

To generate *OsVIRK1* transgenic rice plants, the *OsVIRK1-FLAG-HA*, *OsVIRK1*^*K391E*^*-FLAG-HA*, *OsVIRK1*^*C4A*^*-FLAG-HA*, and *OsVIRK1*^*6A*^*-GFP* coding sequences were cloned separately into the pCAMBIA1300 vector. To generate *OsVIRK1*-knockout transgenic rice plants using the CRISPR/Cas9 method, gRNAs targeting *OsVIRK1* were designed using a web-based tool (http://crispr.dbcls.jp/) and then inserted into the VK005-1 vector. The desired plasmids were introduced into *Agrobacterium tumefaciens* strain EHA105 to transform rice plants (*Oryza sativa* *L*. subsp. *japonica* cv. Nipponbare). To transiently express proteins in *N. benthamiana* leaves, pCAMBIA1300 vectors containing the *OsVIRK1* native promoter-driven *OsVIRK1-GFP* and CaMV 35S promoter-driven *OsVIRK1-GFP*, *OsVIRK1-MYC*, *OsVIRK1-FLAG-HA*, *OsVIRK1N-GFP*, *OsVIRK1C-GFP*, *OsVIRK1*^*K391E*^*-GFP*, *OsVIRK1*^*C4A*^*-GFP*, *OsVIRK1*^*6A*^*-GFP*, *CP*, *CP-HA*, *CP*^*∆1-47*^*-HA*, *CP*^R77A/T79A/D80A/K87A^*-HA*, *CP*^*R122A*^*-HA*, *NS3*^*S117D*^*-GFP*, *NS3*^*T118D*^*-GFP*, *NS3*^*T133D*^*-GFP*, *NS3*^*S182D*^*-GFP*, *NS3*^*S188D*^*-GFP*, *FLAG-HA-NS3*, *FLAG-HA-NS3*^5D^, *SP*, *CRK10-GFP*, *CRK46-GFP*, *AtLYK4-FLAG-HA*, and *AtLYK5-FLAG-HA* were constructed separately. To transiently express proteins in rice protoplasts, *OsVIRK1-GFP*, *OsVIRK1*^*K391E*^*-GFP*, *OsVIRK1*^*C4A*^*-GFP*, *CP*, and *CRK10-GFP* were separately cloned into the pBI221 vector downstream of the CaMV 35S promoter or the *OsVIRK1* native promoter. For BiFC assays, *OsVIRK1*, *CP*, and *NS3* coding sequences were cloned into the pSCYCE or pSCYNE(R) vector^60^. For recombinant protein expression in *E. coli*, *OsVIRK1*, *OsVIRK1N*, *OsVIRK1C*, *CP*, *NS3*, and *SP* were separately cloned into the pET32a, pGEX-3X, or pMAL-p2X vector to express OsVIRK1-His, OsVIRK1N-His, OsVIRK1C-His, CP-GST, NS3-GST, SP-GST, MBP-OsVIRK1C, and MBP-OsVIRK1C^K391E^ fusion proteins. Primers and gRNAs are listed in Supplementary Table S4.

### RSV infection assay

RSV inoculation was performed as described previously using the viruliferous insect (*Laodelphax striatellus*) feeding method^60^. In brief, 9 rice seedlings grown for 10 days in a single pot were exposed to viruliferous or virus-free (for inoculation control) insects at a plant/insect ratio of 1:3. After 3 days of feeding, the insects were removed and the seedlings continued to grow for sample collection and observation of disease symptoms at 28 °C during the day and 22 °C at night with a 16/8-h light/dark photoperiod.

### RNA extraction and RT-qPCR analysis

Total RNA was extracted from plant samples using the AG RNAex Pro Reagent (Accurate Biology). Total RNA (2 μg) was reverse transcribed using the Evo M-MLV Mix Kit with gDNA Clean according to the manufacturer’s protocol (Accurate Biology). The cDNA products were used for quantitative PCR assays with gene-specific primers using the SYBR Green Premix Pro Taq HS qPCR Kit (Accurate Biology) and a Bio-Rad real-time PCR detection system. Relative gene expression levels were quantified using the 2^−∆∆Ct^ method, normalized to the amount of *OsActin1* cDNA detected in the same sample. The primers are listed in Supplementary Table S4.

### Transient expression in rice protoplasts

Rice protoplasts were isolated as described previously^60^. In brief, the sheaths and stems of 2-week-old rice seedlings were cut into 0.5-mm strips and soaked in cell-wall digestion buffer (1.5% cellulase RS, 0.75% macerozyme R-10, 0.6 M mannitol, 10 mM MES, pH 5.7, 10 mM CaCl_2_, 0.1% BSA) for 6 h at 25 °C in the dark with gentle shaking (45–60 rpm). After digestion, an equal volume of W5 solution (154 mM NaCl, 125 mM CaCl_2_, 5 mM KCl, and 2 mM MES, pH 5.7) was added. The protoplast pellets were then collected by filtering through nylon mesh (70 μm), followed by centrifugation at 200× *g* for 3 min. After washing with an equal volume of W5 solution, the protoplast pellets were resuspended in MMG solution (0.4 M mannitol, 15 mM MgCl_2_, and 4 mM MES, pH 5.7) for transient transfection assays.

Protoplast transfection was performed using the PEG-mediated method^60^. In brief, 5–10 μg of plasmids were mixed with 100 μL of protoplasts. After addition of equal volumes of PEG solution (40% (w/v) PEG 4000) (Sigma-Aldrich), 0.2 M mannitol, and 0.1 M CaCl_2_, the mixture was incubated at room temperature for 10–15 min in the dark. A two-fold volume of W5 solution was then added to the mixture. Protoplasts were collected by centrifugation (200× *g* for 3 min) and resuspended in W5 solution. After 16 h of cultivation at 25 °C in the dark, the protoplasts were used for observation of protein subcellular localization with a confocal laser microscope (Leica Model TCS SP8) or for extraction of total RNA for RT-qPCR.

### Transient expression in *N. benthamiana* leaves

Transient expression in *N. benthamiana* leaves was performed as described previously^60^. In brief, *A. tumefaciens* (EHA105) transformants harboring the indicated constructs were grown overnight in culture containing 50 μg/mL kanamycin, 10 mM MES, and 20 mM acetosyringone. Agrobacterial cells were harvested by centrifugation and resuspended in MMA buffer (10 mM MgCl_2_, 10 mM MES, pH 5.6, and 100 mM acetosyringone) to an optical density. After incubation for 3 h at room temperature, an agrobacterial cell suspension containing the indicated construct or combination of constructs was pressure-infiltrated into *N. benthamiana* leaves. Leaves were harvested after 2 days for observation of protein subcellular localization using a confocal laser microscope (Leica Model TCS SP8) or for extraction of total protein for western blot and co-IP assays.

### Membrane fractionation assay

Membrane fractions were isolated using the Membrane Fraction Extraction Kit (Invent Biotechnologies, SM-005-P) as described previously^61^. In brief, protoplasts from *OsVIRK1-FLAG-HA-*overexpressing transgenic rice seedlings were isolated and transformed with *SCAMP1-RFP*. The rice protoplasts were lysed to isolate membrane fractions by gradient centrifugation. First, the intact nuclei and large debris pellet were discarded after centrifugation at 700× *g* for 1 min; then, the supernatant containing cytoplasmic components was discarded after centrifugation at 16,000× *g* for 30 min. The total membrane protein pellet was then gently resuspended to isolate vesicle proteins and PM proteins. After organelle removal by centrifugation at 7800× *g* for 5 min, the sample was centrifuged at 16,000× *g* for 30 min to obtain the supernatant containing vesicle proteins and the pellet containing PM proteins. The total membrane, PM, and vesicle protein samples were subjected to western blot analysis using anti-HA, anti-RFP, or anti-H^+^-ATPase antibodies.

### Western blot assay

Plant samples were ground to a fine powder in liquid nitrogen and homogenized in protein extraction buffer (50 mM Tris-HCl, pH 7.5, 150 mM NaCl, 2 mM EDTA, 50% glycerol, 1% Triton X-100, 1 mM DTT, 1 mM PMSF, 100 μM MG132, 1× protease inhibitor cocktail). After centrifugation at 12,000× *g* at 4 °C, the supernatant was collected as total protein, then separated by SDS-PAGE and electrophoretically transferred to a polyvinylidene difluoride membrane (Amersham Bioscience) for western blot analysis using antibodies against GFP (Abcam), FLAG (Sigma), HA (Abcam), MYC (Easybio), 6× His, CP, or SP.

To examine protein redox regulation, total protein was extracted using extraction buffer with or without DTT. The protein extracts were separated by SDS-PAGE under non-reducing conditions for western blot assays.

### Co-IP assay

Total protein was extracted from the desired plant samples, and anti-GFP, anti-FLAG, or anti-HA beads were pre-equilibrated and added to the total protein supernatant to perform co-IP assays. After gentle shaking at 4 °C for 3 h, the beads were collected by centrifugation at 500× *g* for 2 min, then washed 3 times with protein extraction buffer and subjected to western blot analysis using the desired antibodies.

### Pull-down assay

For GST and His pull-down assays, OsVIRK1-His, OsVIRK1N-His, OsVIRK1C-His, CP-GST, NS3-GST, and SP-GST fusion proteins and control proteins were expressed from recombinant plasmid-harboring *E. coli* BL21 (DE3) and purified using Ni-NTA Superflow agarose (QIAGEN) and glutathione Sepharose 4B (GE Healthcare) according to the manufacturer’s instructions. Mixtures of the designated purified proteins were incubated with glutathione Sepharose beads or Ni-NTA beads in pull-down buffer (20 mM Tris-HCl, pH 7.5, 100 mM NaCl, 1 mM EDTA, 1 mM DTT, 1× protease inhibitor) at 4 °C for 2 h. After centrifugation and washing 3–5 times with wash buffer (20 mM Tris-HCl, pH 7.5, 300 mM NaCl, 0.5% Triton X-100, 1 mM EDTA, 1 mM DTT), the protein-bound beads were loaded onto an SDS-PAGE gel for western blot analysis using antibodies against 6× His and GST.

### MST assay

For MST assays, the OsVIRK1N-His, OsVIRK1C-His, CP-GST, and NS3-GST fusion proteins and control GST protein were expressed from recombinant plasmid-harboring *E. coli* BL21 (DE3) and purified using Ni-NTA Superflow agarose (QIAGEN) and glutathione Sepharose 4B (GE Healthcare) according to the manufacturer’s instructions. The purified proteins were concentrated with 50-kDa Merck Millipore filters and solubilized with HEPES buffer (54.55 mM HEPES, 275 mM NaCl, 1.48 mM Na_2_HPO_4_). Then, 200 nM purified OsVIRK1N-His or OsVIRK1C-His protein was labeled with 100 nM labeling dye according to the manufacturer’s instructions (NanoTemper). The labeled proteins were mixed with gradient-diluted CP-GST, NS3-GST, or GST protein at concentrations from 0.002 μM to 150 μM. After centrifugation at 14,000 rpm and 4 °C for 10 min, the supernatants were loaded into capillaries (NanoTemper), and MST analysis was performed using a Monolith NT.115 instrument (NanoTemper Technologies) at 80% MST power and 20% excitation power. Raw data were analyzed using MO Affinity Analysis software (v2.2.4). All experiments were repeated at least three times.

### In vitro kinase assay

To test the kinase activity of OsVIRK1, the cytoplasmic kinase region of OsVIRK1 fused to the MBP tag (MBP-VIRK1C) was expressed and purified from *E. coli*. As a negative control, the highly conserved Lys residue (K391) required for CRK kinase activity in the ATP-binding domain of OsVIRK1 was mutated to a Glu residue (E391) to produce MBP-VIRK1C^K391E^ purified protein. The two fusion proteins were subjected to in vitro kinase activity assays by incubation with 3 μCi [γ-^32^P] ATP in kinase assay buffer (50 mM HEPES, pH 7.5, 10 mM MgCl_2_, 50 mM NaCl, 100 μM unlabeled ATP, and 1 mM DTT) at 22 °C. The reactions were stopped at different time points (2 min, 10 min, 30 min, and 60 min). To test whether CP or NS3 could be phosphorylated by OsVIRK1, purified recombinant CP-GST or NS3-GST protein was co-incubated with MBP-VIRK1C with 3 μCi [γ-^32^P] ATP in kinase assay buffer. The reactions were stopped after 30 min. Radioactive protein bands were detected by 10% SDS-PAGE followed by autoradiography.

### In vivo phosphorylation assay

For in vivo phosphorylation analysis, *OsVIRK1-GFP*, *OsVIRK1*^*K391E*^*-GFP*, and *OsVIRK1*^*6A*^*-GFP* were transiently expressed in rice protoplasts and *N. benthamiana* leaves, and total proteins were extracted using a buffer containing 50 mM Tris-HCl, pH 7.5, 150 mM NaCl, 10% glycerol, 0.5% Triton X-100, 1 mM PMSF, 1% protease inhibitor cocktail (Sigma), and 2× protein phosphatase inhibitor (ZOMANBIO). OsVIRK1-GFP, OsVIRK1^K391E^-GFP, and OsVIRK1^6A^-GFP were then immunoprecipitated using GFP agarose beads (LAB Lead) and immunoblotted using anti-phosphothreonine (Abcam, ab218195) and anti-phosphoserine (Abcam, ab9332) antibodies. To detect the phosphorylation of OsVIRK1 and NS3 in RSV-infected rice, WT, OsVIRK1-FLAG-HA-OE, and *osvirk1* rice plants were infected with RSV. Total proteins were extracted from mock-treated and RSV-infected rice plants for Phos-tag SDS-PAGE coupled immunoblotting assays using anti-FLAG or anti-NS3 antibodies.

### HPLC-MS/MS analysis

To identify the phosphorylation sites of OsVIRK1C and NS3, the phosphorylation reaction mixtures were separated by 10% SDS-PAGE, then stained with Coomassie Brilliant Blue. The OsVIRK1C or NS3 band from different phosphorylation reactions was excised from the stained gels and subjected to HPLC-MS/MS analysis at QLBio Company (Beijing, China).

### RNA-seq analysis

Total RNA was extracted from WT and *osvirk1* rice protoplasts transfected with CP-harboring plasmids or EV control plasmids. RNA sequencing libraries were constructed and sequenced by BerryGenomics on an Illumina platform using standard protocols (Beijing, China). After removal of adapters and low-quality reads, clean reads were aligned to the reference genome (http://rice.uga.edu/) to analyze differential gene expression. Genes with log_2_(fold change) ≥ 1 and *P* value < 0.05 were considered to be differentially expressed.

### VSR activity assay

The VSR activity assays were conducted using the *GFP* transgenic 16c *N. benthamiana* system as described previously^43^. In brief, *Agrobacterium* strain GV3101 carrying 35S::*GFP* and the designated construct to express OsVIRK1-MYC, FLAG-HA-NS3, FLAG-HA-NS3^5D^, NS3^S117D^, NS3^T118D^, NS3^T133D^, NS3^S182D^, or NS3^S188D^ was co-infiltrated into 16c *N. benthamiana* leaves. Five days after *Agrobacterium* infiltration, GFP fluorescence signals were visualized using a hand-held long-wavelength UV lamp (Blak-Ray B-100AP, Ultraviolet Products), and levels of GFP, OsVIRK1-MYC, FLAG-HA-NS3, and FLAG-HA-NS3^5D^ proteins were detected by western blot assays using the corresponding antibodies.

## Supplementary information

Supplementary information

Supplementary Table S2

## Data Availability

All data are available within this article and its Supplementary information. Source data are provided in Supplementary Table S2.
