# Supplementary material for: A receptor-like kinase recognizes viral proteins at the trans-Golgi network/early endosome and inhibits infection in rice
Source: Cell Discov. 2025 Dec 16;11:101. doi: 10.1038/s41421-025-00847-4 (PMC12708648; doi:10.1038/s41421-025-00847-4)
Supplement: Supplementary file 1 — Supplementary information [file 41421_2025_847_MOESM1_ESM.pdf]

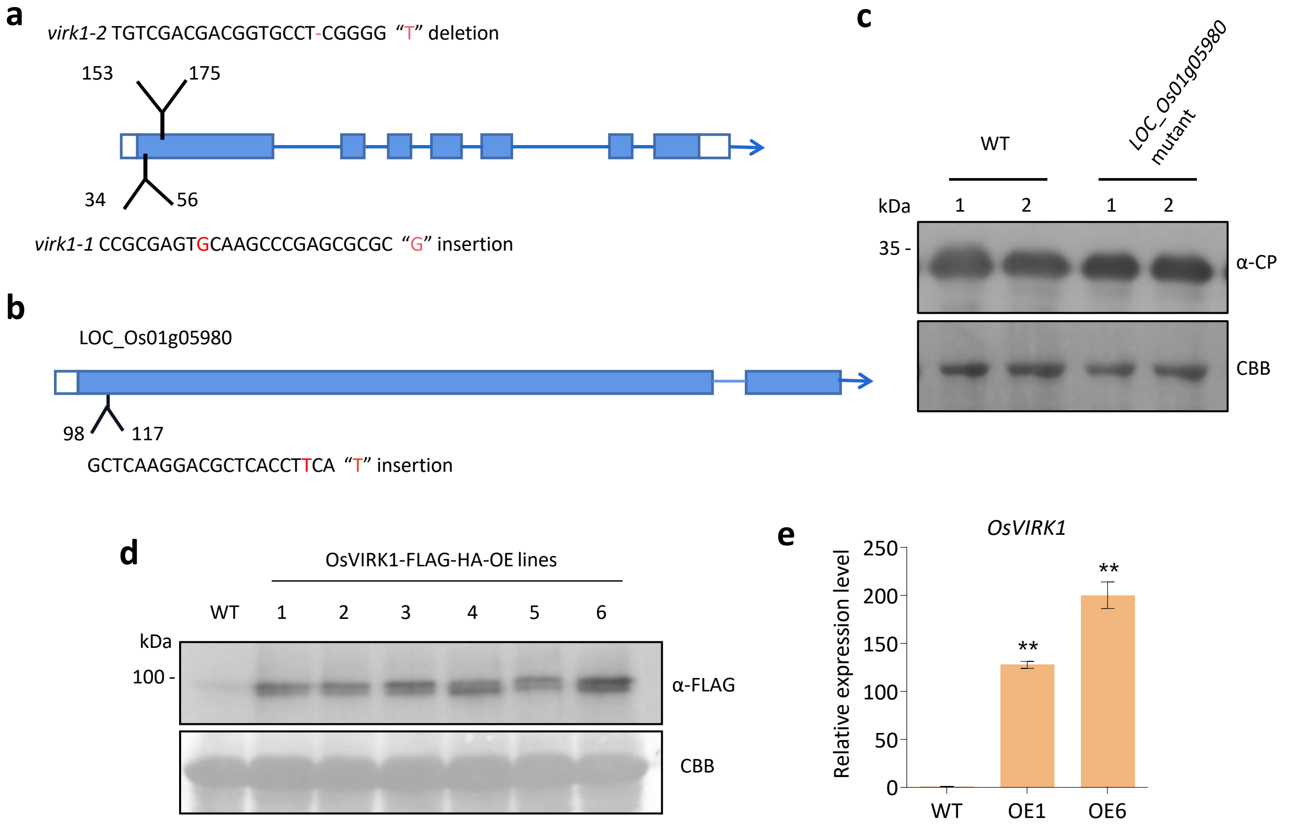

Supplementary Fig. S1 Identification of *Osvirk1* knockout mutants and *OsVIRK1*-overexpressed rice plants. a, Generation of *Osvirk1* knockout mutants by CRISPR-Cas9. Two mutations, *virk1-1* with a "G" insertion and *virk1-2* with a "T" deletion, are indicated. **b,** Generation of the knockout mutant of *LOC_Os01g05980* by CRISPR-Cas9. A mutation of *LOC_Os01g05980* with a "T" insertion is indicated. In a and b, the gene structures are shown with blue and white boxes representing exons and untranslated regions, respectively. **c,** Anti-CP Western blot showing that the knockout mutant of *LOC_Os01g05980* did not show altered CP accumulation upon RSV infection comparing to WT. The numbers 1 and 2 represent the biological replicates. **d,** Anti-FLAG Western blot showing protein levels of OsVIRK1-FLAG-HA in 35S:*OsVIRK1-FLAG-HA* transgenic rice lines. In c and d, the rubisco large subunit bands were visualized by Coomassie Brilliant Blue (CBB) and served as a loading control. **e,** RT-qPCR analysis showing the expression levels of *OsVIRK1* in two 35S:*OsVIRK1-FLAG-HA* transgenic rice lines (OE1 and OE6). Values are mean ± SD (n = 3), $^{**}P < 0.01$, student's *t*-test.

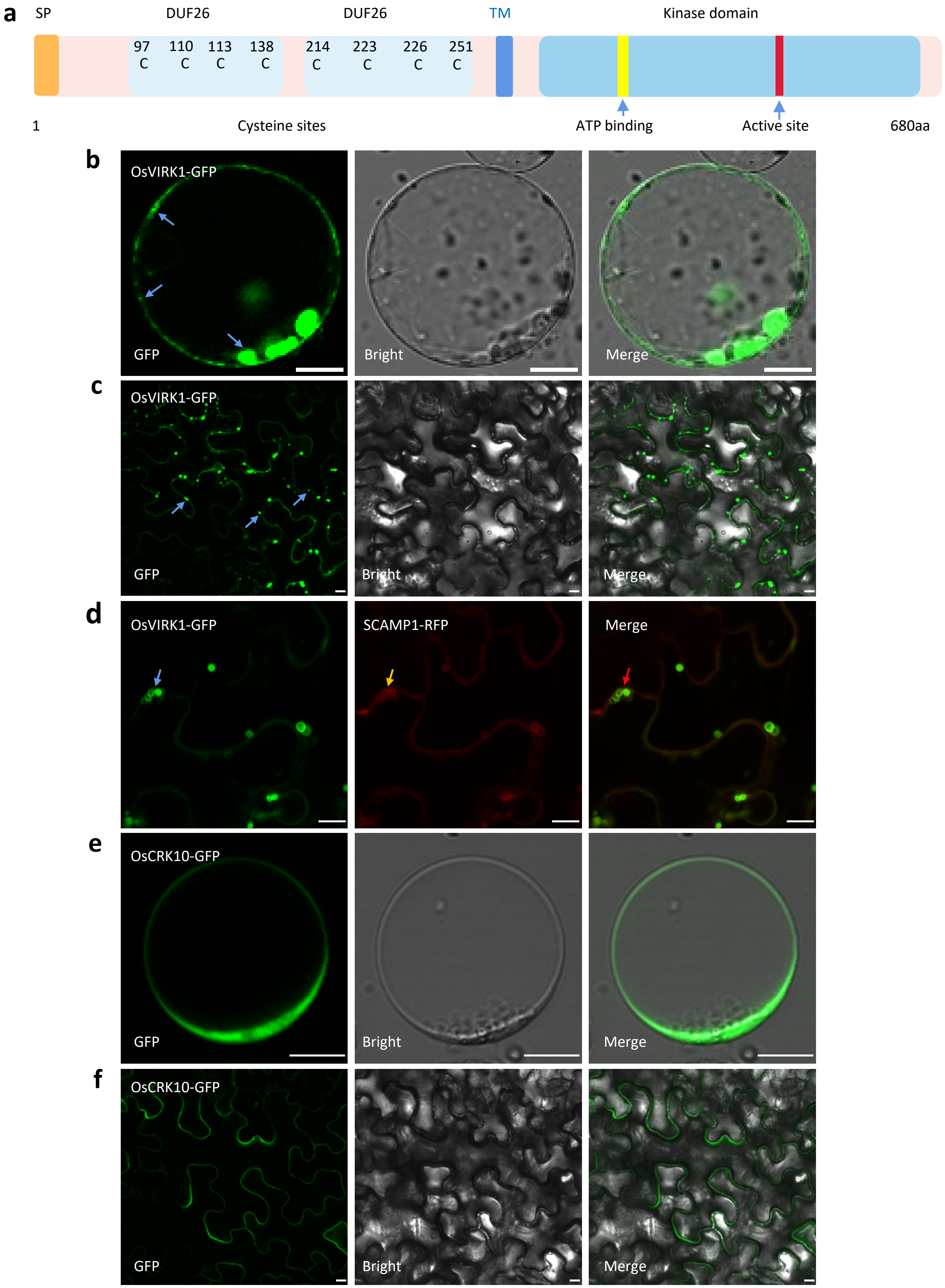

Supplementary Fig. S2 Subcellular localization of OsVIRK1. a, Schematic diagram of the general domain architecture of OsVIRK1 including an N-terminal signal peptide (SP), two predicted DUF26 domains with conserved Cys residues, a transmembrane region (TM), and a kinase domain with conserved regions for ATP binding and kinase activity. **b,c** Subcellular localization analysis of OsVIRK1. OsVIRK1-GFP was transiently expressed in rice protoplasts and *N. benthamiana* leaves, then the GFP fluorescence was observed by confocal laser microscope. **d,** Close observation showing that OsVIRK1-GFP co-localizes with SCAMP1-RFP at the membrane-enveloped bodies. **e,f,** OsCRK10-GFP was transiently expressed in rice protoplasts and *N. benthamiana* leaves, and the GFP fluorescence was observed by confocal laser microscope. In b-d, the arrows indicate the examples of puncta formed by OsVIRK1-GFP and SCAMP1-RFP. Scale bars, 10 μm.

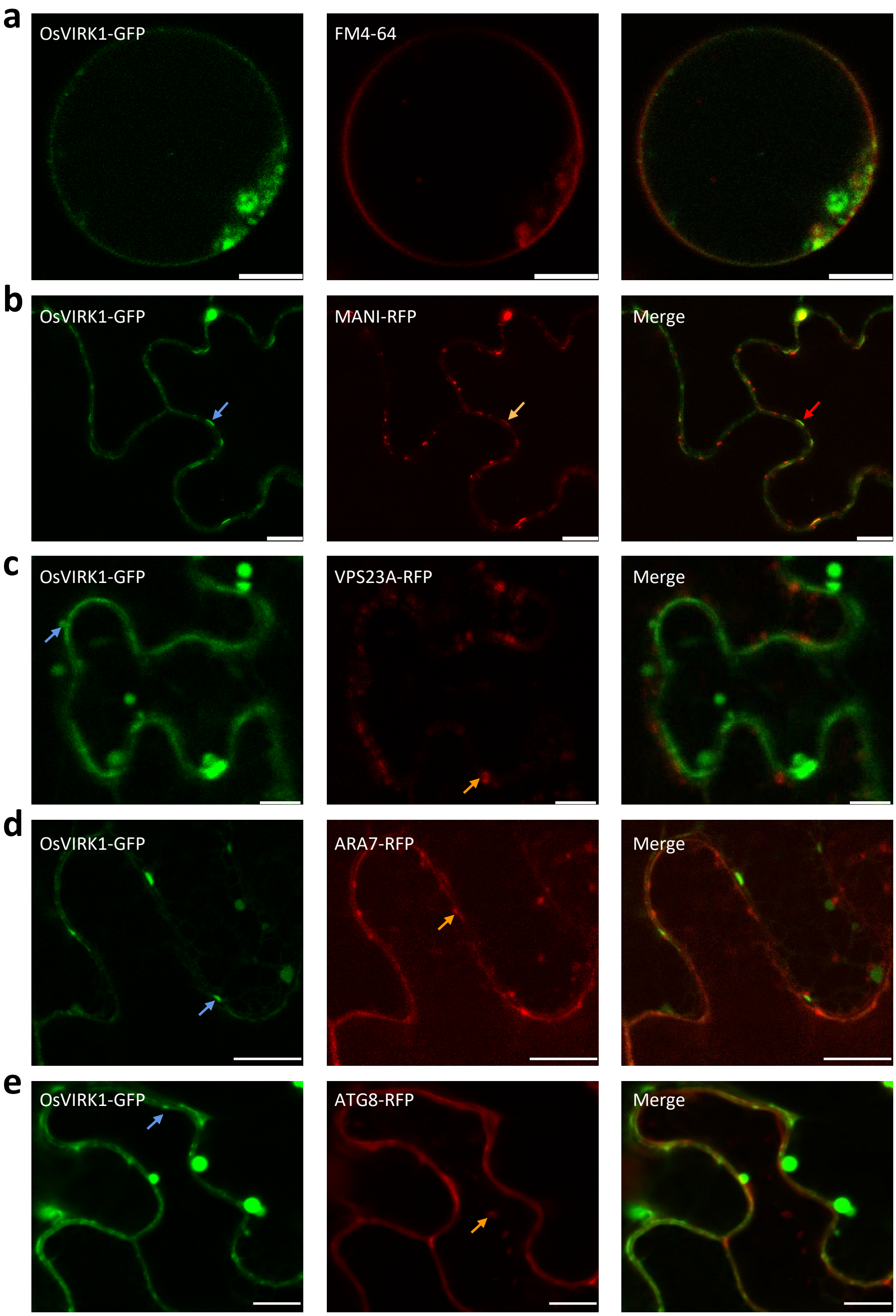

Supplementary Fig. S3 Co-localization analysis of OsVIRK1 with the plasma membrane-associated marker dye FM4-64 and the endomembrane marker proteins. a, The rice protoplast expressing OsVIRK1-GFP was incubated with 2 μM FM4-64. **b-e,** Co-expression of OsVIRK1-GFP with the Golgi marker MANI-RFP (b), the ESCRT-I marker VPS23A-RFP (c), the LE marker ARA7-RFP (d), or the autophagy marker ATG8-RFP (e) in *N. benthamiana* leaves. In a-e, the corresponding fluorescence signal was observed by confocal laser microscope. The blue arrows indicate the examples of OsVIRK1-GFP formed puncta; the yellow arrows indicate the typical example of MANI-RFP-, FM4-64, VPS23A-RFP-, ARA7-RFP-, or ATG8-RFP-represented body; the red arrow indicates the merged signal. Scale bars, 10 μm.

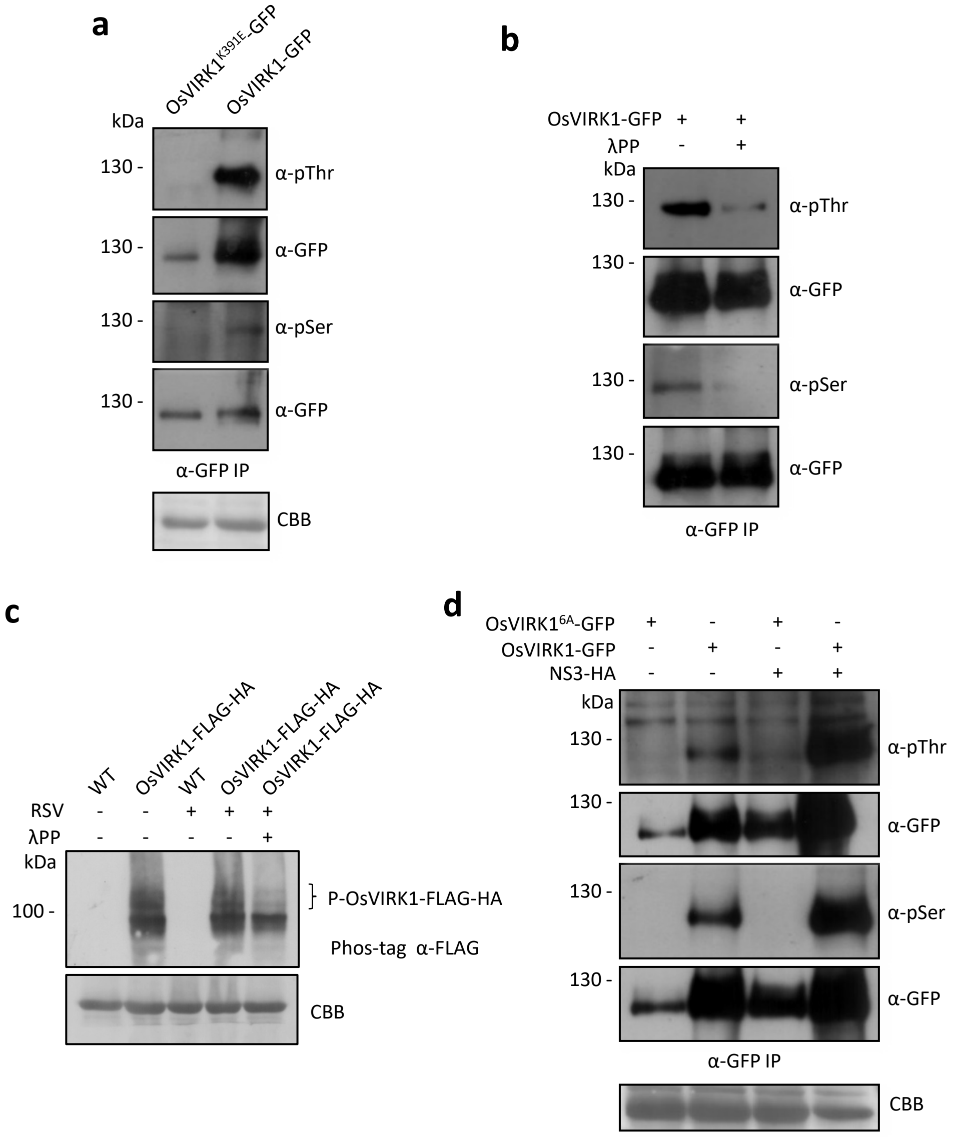

Supplementary Fig. S4 Autophosphorylation analysis of OsVIRK1 in *N. benthamiana*. **a,b,** OsVIRK1-GFP and OsVIRK1^{K391E}-GFP were transiently expressed in *N. benthamiana* leaves, then total proteins were extracted to perform anti-GFP immunoprecipitation and subsequent anti-pThr and anti-pSer Western blot with or without λPP treatment. **c,** Phos-tag SDS-PAGE coupled immunoblotting assay showing that OsVIRK1-FLAG-HA is phosphorylated in VIRK1-FLAG-HA-OE rice plants with or without RSV infection. **d,** OsVIRK1-GFP and OsVIRK1^{6A}-GFP were transiently expressed in *N. benthamiana* leaves with or without coexpressing NS3, then total proteins were extracted to perform anti-GFP immunoprecipitation and subsequent anti-pThr and anti-pSer Western blot. All of the experiments were repeated two or three times with similar results.

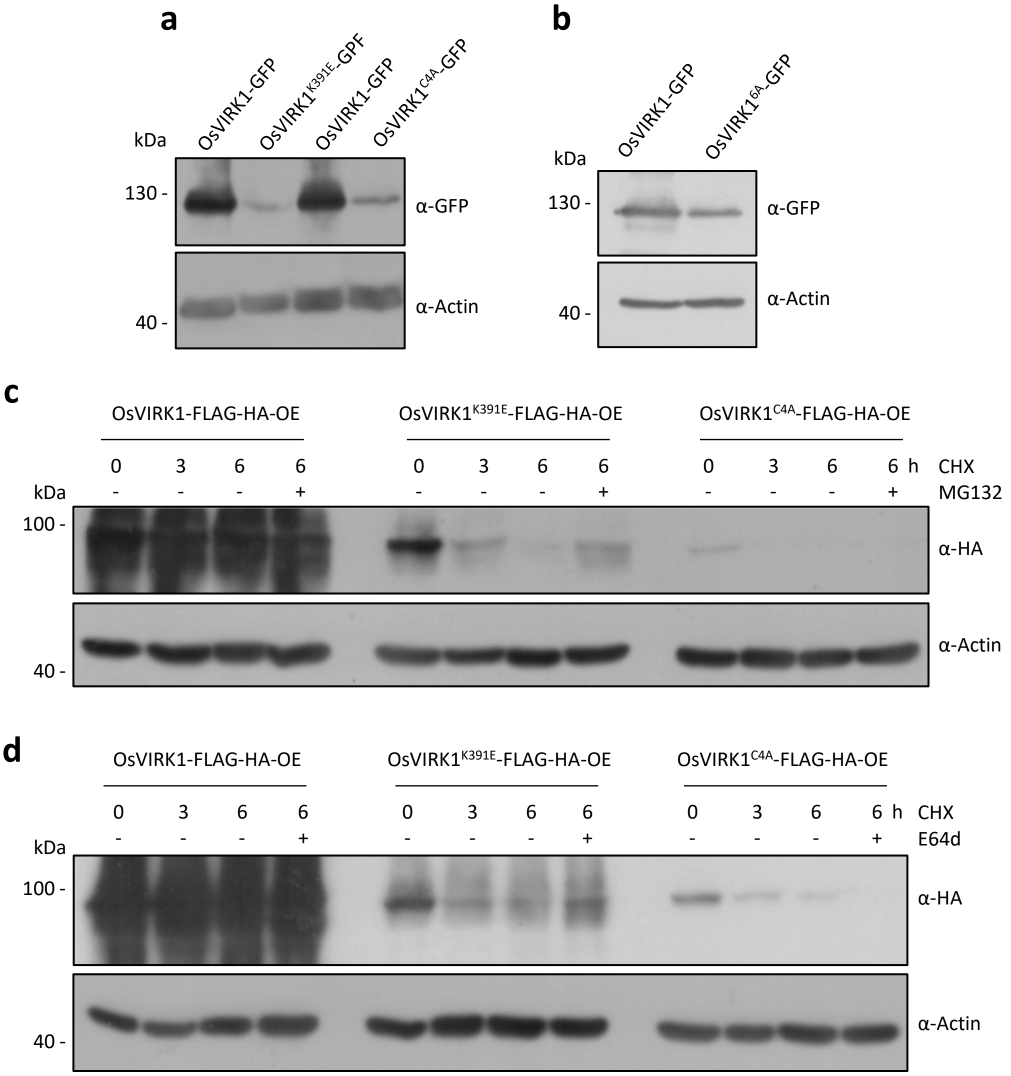

Supplementary Fig. S5 The autophosphorylation and redox regulation of OsVIRK1 confer its accumulation. a, Anti-GFP Western blot showing the protein levels of OsVIRK1-GFP, OsVIRK1^{K391E}-GFP and OsVIRK1^{C4A}-GFP when they were transiently expressed in *N. benthamiana* leaves. **b,** Anti-GFP Western blot showing the protein levels of OsVIRK1-GFP and OsVIRK1^{6A}-GPF when they were transiently expressed in *N. benthamiana* leaves. **c,d,** Protein degradation analysis of OsVIRK1-FLAG-HA, OsVIRK1^{K391E}-FLAG-HA and OsVIRK1^{C4A}-FLAG-HA. Total proteins were extracted from the 100 mM CHX-treated transgenic rice plants of OsVIRK1-FLAG-HA-OE, OsVIRK1^{K391E}-FLAG-HA-OE and OsVIRK1^{C4A}-FLAG-HA-OE with or without the addition of MG132 or E64d, then the protein accumulation was examined using anti-HA Western blot. All of the experiments were repeated two or three times with similar results.

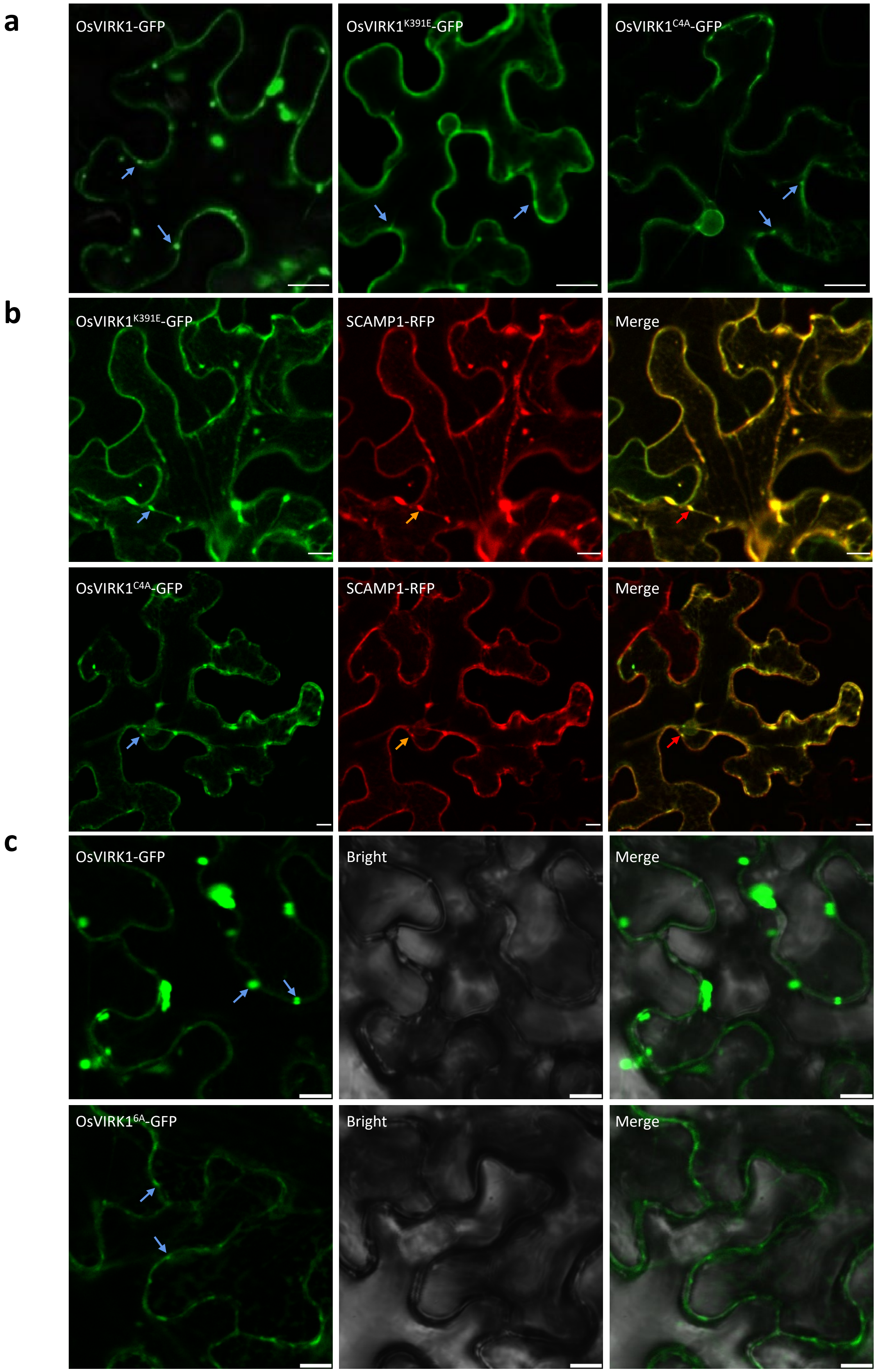

Supplementary Fig. S6 Localization analysis of OsVIRK1-GFP, OsVIRK1^{K391E}-GFP, OsVIRK1^{C4A}-GFP, and OsVIRK1^{6A}-GFP. a, Subcellular localization of OsVIRK1-GFP, OsVIRK1^{K391E}-GFP and OsVIRK1^{C4A}-GFP proteins in *N. benthamiana* leaves. **b,** Co-expression of OsVIRK1^{K391E}-GFP and OsVIRK1^{C4A}-GFP with the TGN/EE marker SCAMP1-RFP in *N. benthamiana* leaves. **c,** Subcellular localization of VIRK1-GFP and VIRK1^{6A}-GFP proteins in *N. benthamiana* leaves. The corresponding fluorescence was observed by confocal laser microscope. The arrows indicate the examples of puncta formed by OsVIRK1-GFP, OsVIRK1^{K391E}-GFP, OsVIRK1^{C4A}-GFP, OsVIRK1^{6A}-GFP, SCAMP1-RFP or merged signal. Scale bars, 10 μm.

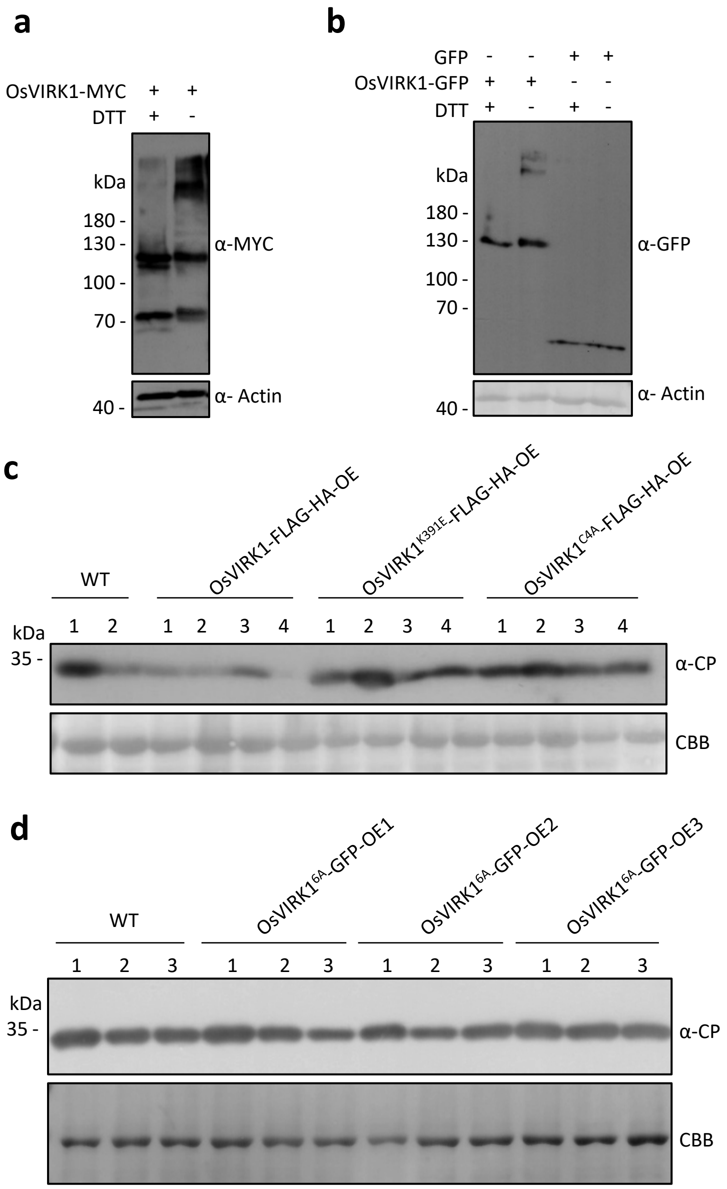

Supplementary Fig. S7 The kinase active site and the conserved Cys residues of OsVIRK1 affect its antiviral function. **a,b,** Analysis of the redox regulation of OsVIRK1. OsVIRK1-MYC and OsVIRK1-GFP were transiently expressed in *N. benthamiana* leaves. Then, the total proteins were extracted with or without DTT (10 mM) and were separated by non-reducing SDS-PAGE followed by Western blot analysis using anti-GFP and -MYC antibodies. **c,** Western blot showing the accumulation of CP in RSV-infected WT and transgenic rice plants overexpressing of OsVIRK1-FLAG-HA, OsVIRK1^{K391E}-FLAG-HA and OsVIRK1^{C4A}-FLAG-HA. **d,** Western blot showing the accumulation of CP in RSV-infected WT and transgenic rice plants overexpressing of *OsVIRK1*^{6A}*-GFP*. In a and b, anti-actin Western blot analysis was used as the loading control. In c and d, the numbers (1, 2, 3, 4) represent the biological replicates, the rubisco large subunit bands were visualized by Coomassie Brilliant Blue (CBB) and served as a loading control. All of the experiments were repeated two or three times with similar results.

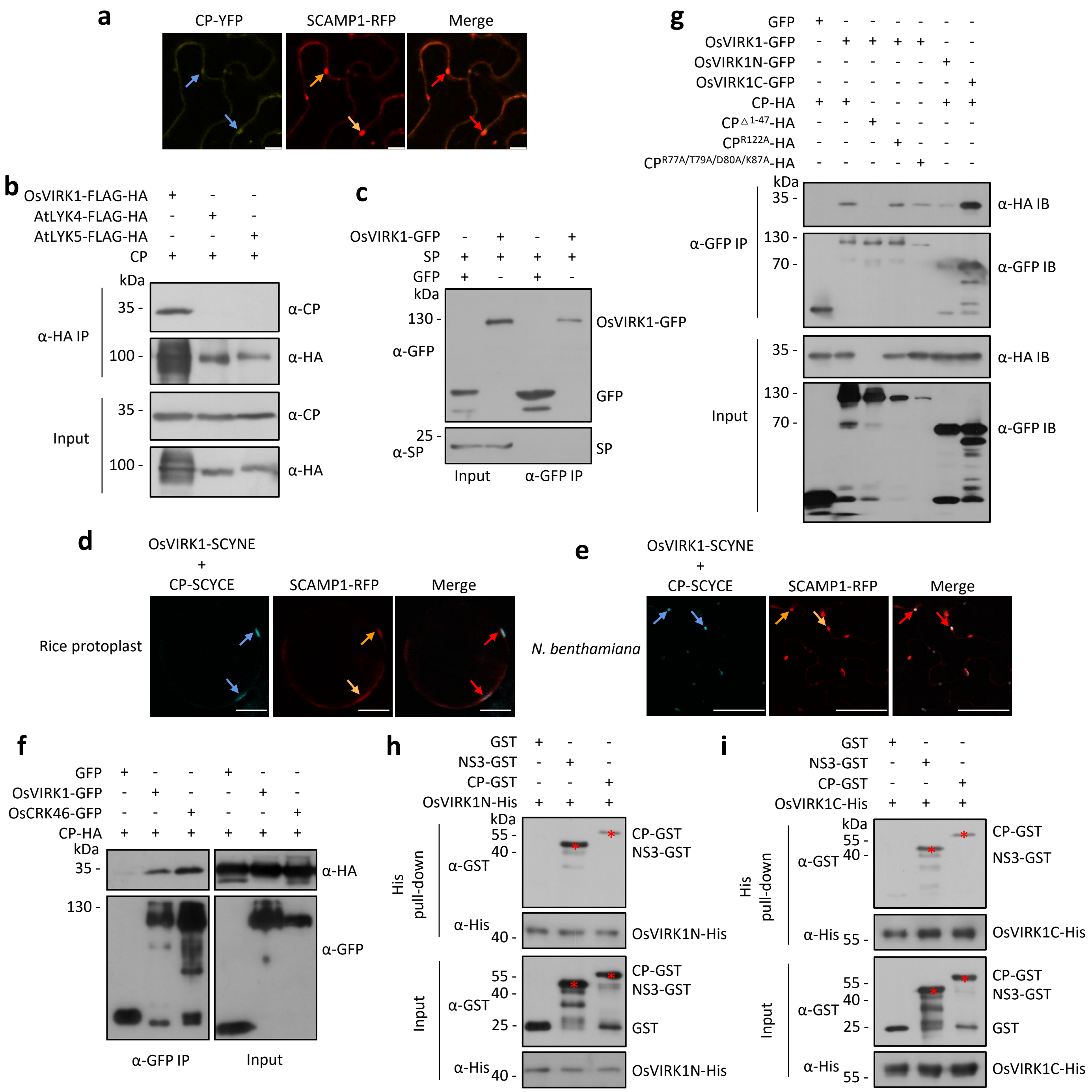

Supplementary Fig. S8 OsVIRK1 interacts with RSV CP at the TGN/EE. a, The puncta of CP-YFP and SCAMP1-RFP partially colocalized when they were coexpressed in *N. benthamiana* leaves. The YFP and RFP fluorescence signals were observed by confocal laser microscope. The arrows indicate the examples of puncta formed by CP-YFP and SCAMP1-RFP. Scale bars are 10 μm. **b,** Co-IP assay showing that CP interacts with OsVIRK1, but not with LYK4 and LYK5 in *N. benthamiana*. **c,** Co-IP assay showing that OsVIRK1 is not associated with SP in *N. benthamiana*. In b and c, the indicated constructs were transiently expressed in *N. benthamiana* leaves, then the proteins were subjected to anti-GFP (b) and anti-HA (c) IP and subsequent Western blot analysis using anti-GFP, -SP, -CP and -HA antibodies. **d,e,** BiFC assay showing that OsVIRK1 interacts with CP at the TGN/EE. The designated construct pairs were cotransfected with the SCAMP1-RFP into rice protoplasts and *N. benthamiana* leaves, then the CFP and RFP fluorescence signals were observed. The arrows indicate the examples of puncta formed by the interaction of OsVIRK1-SCYNE/CP-SCYCE and SCAMP1-RFP. Scale bars are 10 μm (d) or 50 μm (e). **f,** Co-IP assay showing that OsVIRK1 and OsCRK46 interacts with CP. The indicated constructs were transiently expressed in *N. benthamiana* leaves, then the proteins were subjected to anti-GFP IP and subsequent Western blot analysis using anti-GFP and -HA antibodies. **g,** Co-IP assay showing that OsVIRK1, OsVIRK1N and OsVIRK1C interact with CP and CPs mutated in residues required for RNA binding. The indicated constructs were transiently expressed in *N. benthamiana* leaves, then the proteins were subjected to anti-GFP IP and subsequent Western blot analysis using anti-GFP and -HA antibodies. **h,i,** His pull-down assay showing that both the N- and C-terminal regions of OsVIRK1 bind to CP and NS3 *in vitro*. His-tagged OsVIRK1 regions (OsVIRK1N-His and OsVIRK1C-His) and GST-tagged CP (CP-GST) and NS3 (NS3-GST) recombinant proteins were affinity purified, and the protein-protein interaction was tested by His pull-down assays. The red stars mark the expected protein bands in the Western blots. All of the experiments were repeated two or three times with similar results.

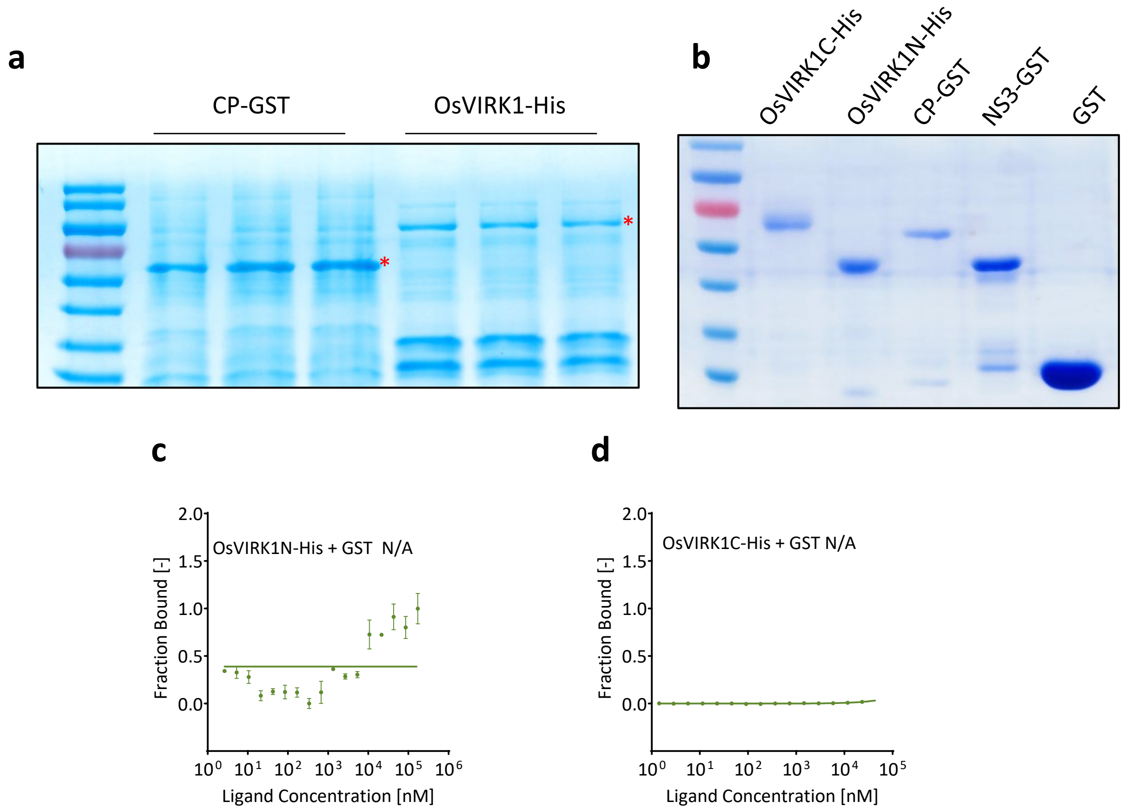

Supplementary Fig. S9 a, Coomassie Brilliant Blue (CBB) staining showing the purified fusion proteins used in His pull down assays. **b,** Coomassie Brilliant Blue (CBB) staining showing the purified fusion proteins used in MST assays. **c,d,** MST analysis showing no binding affinity between OsVIRK1N-His or OsVIRK1C-His and GST. N/A indicates no binding affinity.

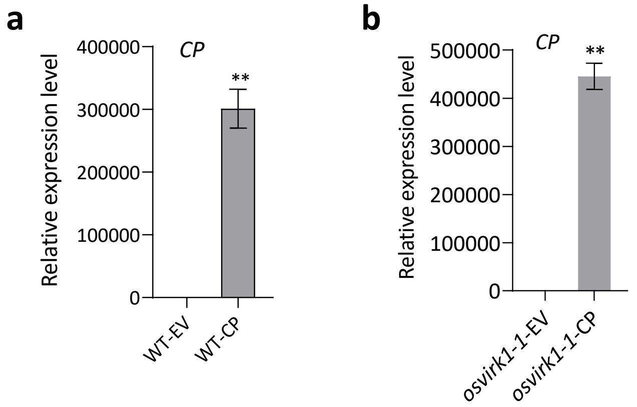

Supplementary Fig. S10 Transient expression of *CP* in WT and *osvirk1-1* rice protoplasts. a,b 35S:*CP* and empty vector (EV, as a control) were transiently expressed in the rice protoplasts of WT and *osvirk1-1* mutant plants, then the expression of *CP* RNA was examined by RT-qPCR. Values are means ±SD (n = 3 biological replicate), $^{**}P < 0.01$, student's *t* test.

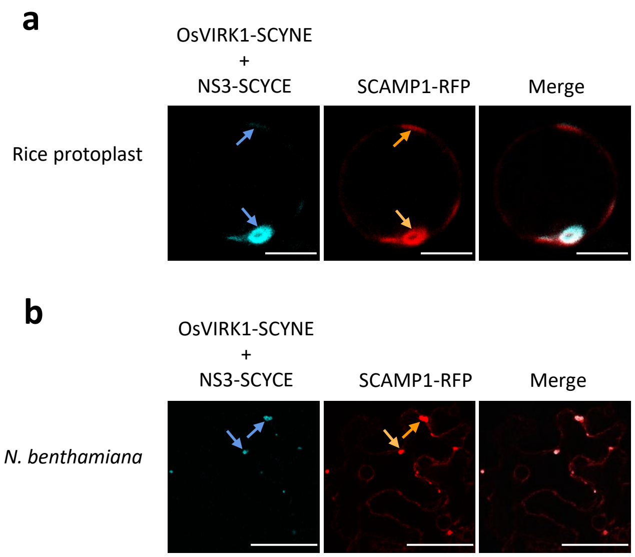

Supplementary Fig. S11 OsVIRK1 interacts with RSV NS3 at the TGN/EE. a,b, BiFC assay showing that OsVIRK1 interacts with NS3 at the TGN/EE. The designated construct pairs were cotransfected with SCAMP1-RFP into rice protoplasts and *N. benthamiana* leaves, then the CFP and RFP fluorescence signals were observed. The arrows indicate the examples of puncta formed by the interaction of OsVIRK1-SCYNE and NS3-SCYCE or SCAMP1-RFP. Scale bars are 10 µm (a) or 50 µm (b). All of the experiments were repeated two or three times with similar results.

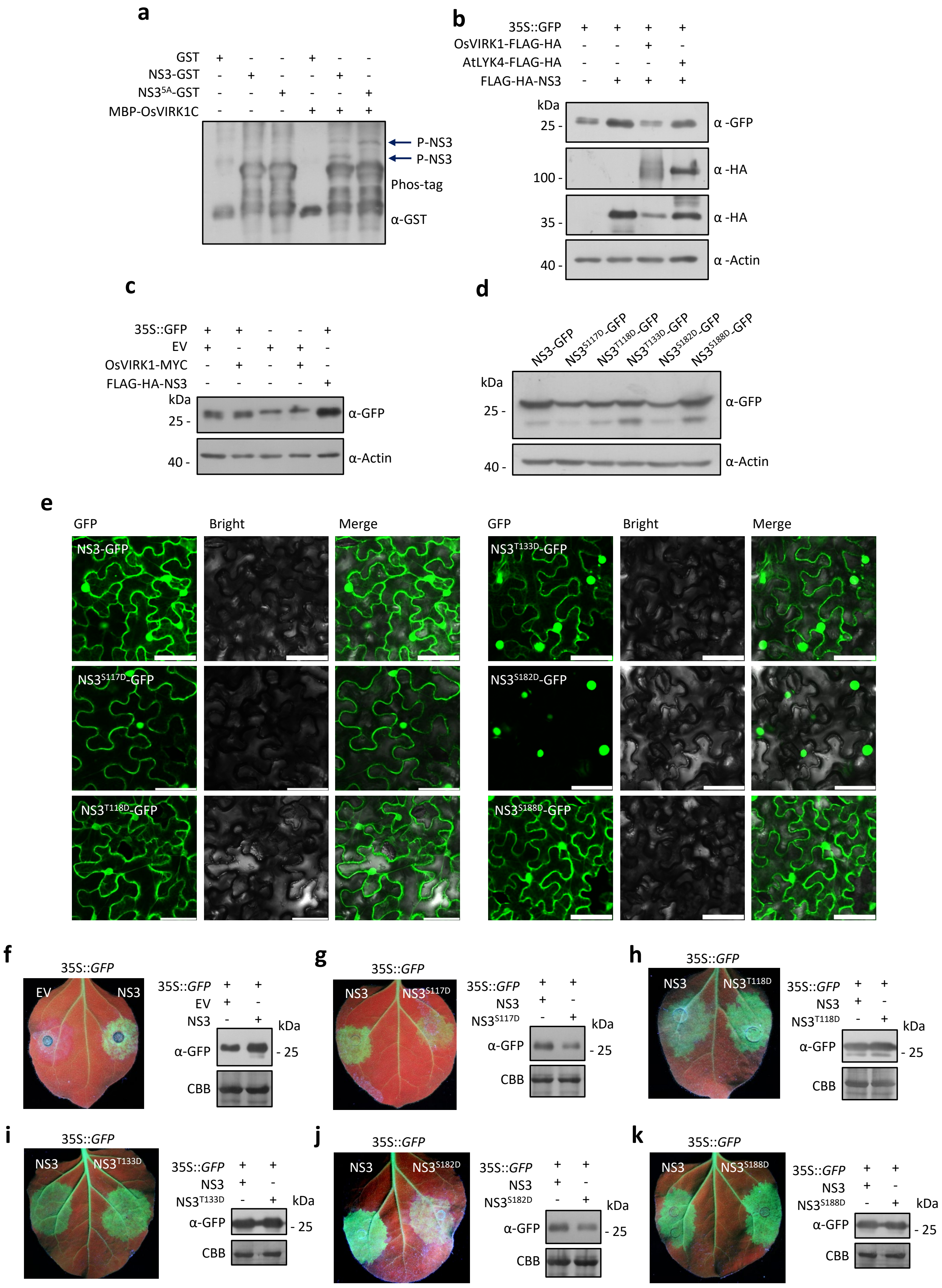

a
GST
NS3-GST
NS3^5A-GST
MBP-OsVIRK1C
P-NS3
P-NS3
Phos-tag
α-GST
b
35S::GFP
OsVIRK1-FLAG-HA
AtLYK4-FLAG-HA
FLAG-HA-NS3
kDa
25 -
100 -
35 -
40 -
α -GFP
α -HA
α -HA
α -Actin
c
35S::GFP
EV
OsVIRK1-MYC
FLAG-HA-NS3
kDa
25 -
40 -
α-GFP
α-Actin
d
NS3-GFP
NS3S117D-GFP
NS3T118D-GFP
NS3T133D-GFP
NS3S182D-GFP
NS3S188D-GFP
kDa
25 -
40 -
α-GFP
α-Actin
e
GFP
Bright
Merge
NS3-GFP
NS3S117D-GFP
NS3T118D-GFP
NS3T133D-GFP
NS3S182D-GFP
NS3S188D-GFP
f
35S::GFP
EV
NS3
NS3
kDa
α-GFP
- 25
CBB
g
NS3S117D
h
NS3T118D
i
NS3T133D
j
NS3S182D
k
NS3S188D

Supplementary Fig. S12 Analysis the effect of the five potential phosphorylation sites on NS3 accumulation and VSR activity. a, Analysis of the phosphorylation of NS3 and $NS3^{5A}$ by OsVIRK1 *in vitro*. The kinase reactions were performed by mixing the desired fusion proteins of OsVIRK1C-MBP, GST, NS3-GST and $NS3^{5A}$-GST, then the reaction products were analyzed by Phos-tag SDS-PAGE Western blot using anti-GST antibody. The arrows point to the phosphorylated bands. **b,** OsVIRK1-FLAG-HA, not AtLYK4-FLAG-HA, reduces the VSR function of NS3. The VSR activity of NS3 was assessed by transient co-expression of 35S::*GFP* and FLAG-HA-NS3 with OsVIRK1-FLAG-HA or AtLYK4-FLAG-HA in 16c leaves. The accumulation of GFP, FLAG-HA-NS3, OsVIRK1-FLAG-HA and AtLYK4-FLAG-HA was analyzed by Western blots. **c,** Examination of the accumulation of GFP when Os*VIRK1* was expressed with or without 35S::*GFP* in 16c leaves. **d,** Examination of the accumulation of NS3-GFP, $NS3^{S117D}$-GFP, $NS3^{T118D}$-GFP, $NS3^{T133D}$-GFP, $NS3^{S182D}$-GFP and $NS3^{S188D}$-GFP by Western blot using anti-GFP antibody when they were transiently expressed in *N. benthamiana* leaves. All of them were expressed in different zones of one leaf. **e,** Subcellular localization of NS3-GFP, $NS3^{S117D}$-GFP, $NS3^{T118D}$-GFP, $NS3^{T133D}$-GFP, $NS3^{S182D}$-GFP and $NS3^{S188D}$-GFP proteins when transiently expressed in *N. benthamiana* leaves. Bars, 50 µm. **f-k,** Examination of the VSR activity of $NS3^{S117D}$, $NS3^{T118D}$, $NS3^{T133D}$, $NS3^{S182D}$ and $NS3^{S188D}$. The VSR activity was assessed by transient co-expression of 35S::*GFP* and EV, NS3, $NS3^{S117D}$, $NS3^{T118D}$, $NS3^{T133D}$, $NS3^{S182D}$ or $NS3^{S188D}$ in 16c leaves. The GFP protein accumulation was analyzed by Western blot using anti-GFP antibody and the GFP fluorescence was imaged 5 days post agroinfiltration. EV, empty vector. All of the experiments were repeated at least two times with similar results.

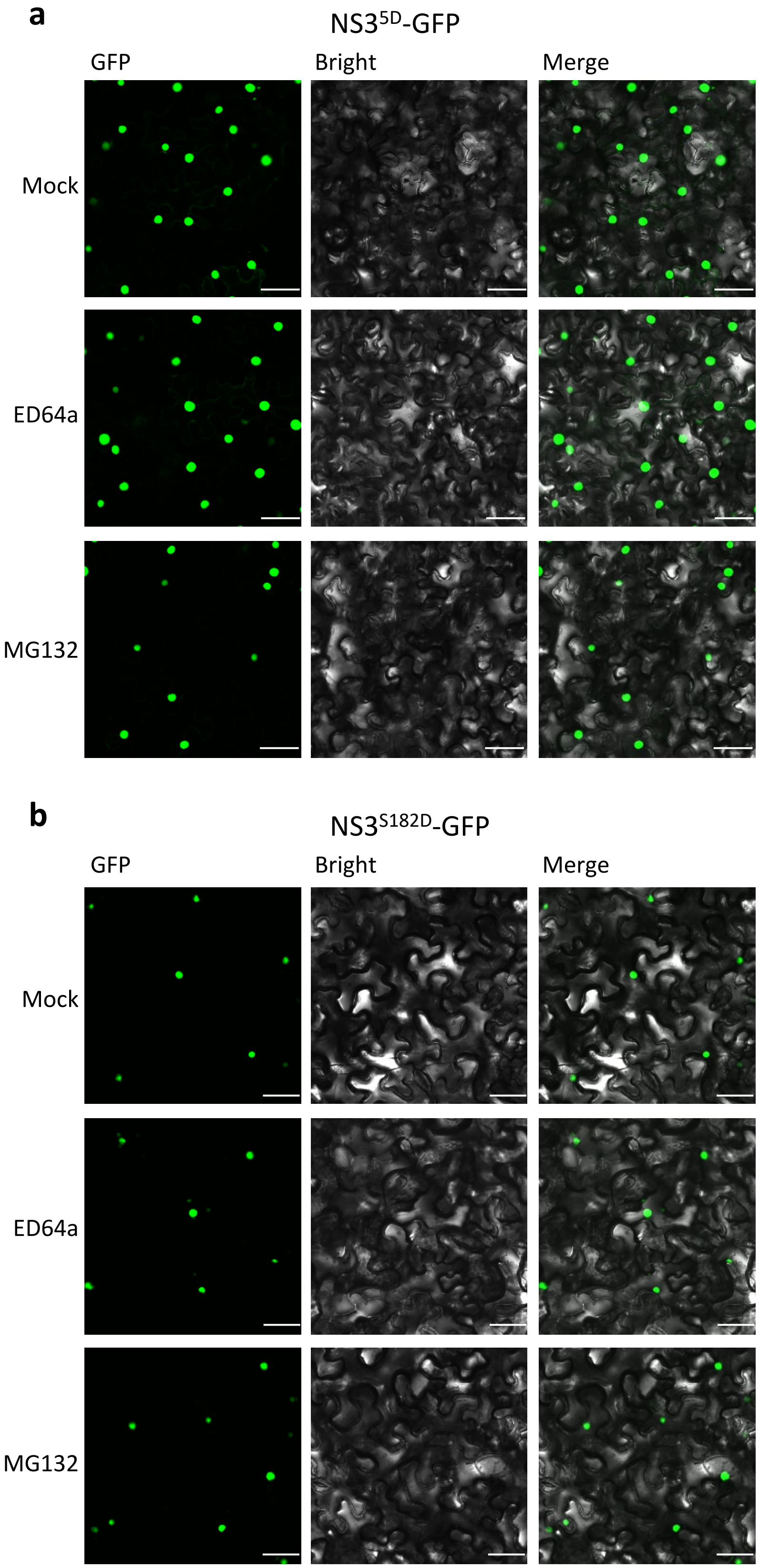

Supplementary Fig. S13 Analysis the effect of ED64a and MG132 on the subcellular location of NS3^{5D} and NS3^{182D}. a,b, NS3^{5D}-GFP and NS3^{S182D}-GFP were transiently expressed in *N. benthamiana* leaves for 12 hours and then treated by mock, ED64a or MG132 for 24 hours to observe the GFP signals. Bars, 50 µm. All of the experiments were repeated at least two times with similar results.

Supplementary Table S1 The potential autophosphorylation sites of OsVIRK1 identified by MS analysis.

Position	Phospho (STY) Probabilities
T360; S364	AAT(1)ANFS(1)EENKLGEGGFGPVYK
T396	RLSAT(0.995)S(0.005)HQGQLEMK
T451	SLDTILFDT(0.988)S(0.012)RQQDLNWEQR
S492	IIHRDLKAS(1)NILLDVDMNPK
T528	IAGT(1)YGYMAPEYALHGIFSAK

Supplementary Table S3 The potential phosphorylation sites of NS3 identified by MS analysis.

Position	Phospho (STY) Probabilities
S117; T118	FFTEVKPRPPS(0.417)T(0.417)NCWT(0.167)CR
T133	MS(0.006)KDNLPFT(0.904)VPS(0.09)VK
S182	Y(0.004)VIS(0.156)S(0.511)DKPPLS(0.329)AR
S188	YVISSDKPPLS(1)AR

Supplementary Table S4 Oligonucleotides used in this study

**Primers (Sequence 5'-3')**	
**Constructs for expression in plants**	
OsVIRK1-FlagHA-F	ACACGGGGGACTATGGCGCCGCCGCC
OsVIRK1-FlagHA-R	TTTGTAGTCCATAGGGTTGATCGACGGT
OsVIRK1-MYC-F	CACGGGGGACTAACACCGCAAC
OsVIRK1-MYC-F	TCGATACCGTCGGTTGATCGACGGT
OsVIRK1-GFP-F	CACGGGGGACTAACACCGCAACAAT
OsVIRK1-GFP-R	TGGTGGCTAGGTTGATCGACGGT
OsVIRK1N-GFP-F	CACGGGGGACTCTAGAATGGCGCCGCCGCCGCCG
OsVIRK1N-GFP-R	GTGGCTAGCGGATCCGCTCGAGCAGCCGTAGGAACCT
OsVIRK1C-GFP-F	GAGAACACGGGGGACTCTAGATGTGCAAGAGGAACAGAAAACC
OsVIRK1C-GFP-R	CCATGGTGGCTAGCGGATCCGCTCGAGCAGCCGTAGGAAC
CP-mcherry-F	ACACGGGGGACTATGGGTACC
CP-mcherry-R	CTCACCATCCCGTCATCTGCAC
CP-HA-F	AGAACACGGGGGACTCTAGAATGGGTACCAACAAGCCAG
CP-HA-R	CGTATCCGCCGCCGGATCCGTCATCTGCACCTTCTGCC
$CP^{\Delta 1\text{-}47}$-HA-F	GAGAACACGGGGGACTCTAGAATGGCTGCAACTCTGATTGG
$CP^{\Delta 1\text{-}47}$-HA-R	CGTATCCGCCGCCGGATCCGTCATCTGCACCTTCTGCC
FlagHA-NS3-F	GAGAACACGGGGGACTCTAGA ATGAACGTGTTCACATCGTC
FlagHA-NS3-R	CATAGGCCTCACGTGTCTAGA CAGCACAGCTGGAGAGC
CRK10-GFP-F	ACACGGGGGACTATGTCCATGGCCTG
CRK10-GFP-R	ATGGTGGCTAGGCTAGCAGTAGCA
**Constructs for expression of recombinant protein**	
OsVIRK1-His-F	GATATCGGATCC AACACCGCAACA
OsVIRK1-His-R	CTCGAGTGCGGCCGCTTGATCGACGGT
OsVIRK1N-His-F	ACGACGACGACAAGGCCATGGATATGGCGCCGCCGCCGCCG
OsVIRK1N-His-R	CTCGAGTGCGGCCGCAAGCTTGGCGTCGAAGAACGGGTAC
OsVIRK1C-His-F	ACGACGACGACAAGGCCATGGATATGTGCAAGAGGAACAGA
OsVIRK1C-His-R	CTCGAGTGCGGCCGCAAGCTTTCGAGCAGCCGTAGGAACC
MBP-OsVIRK1C-F	GAAGGATTTCATGCAAGAGGAAC
MBP-OsVIRK1C-R	CGGCCAGTGCCA GTTGATCGACGG
CP-GST-F	TGATCGAAGGTCGTGGGATCCTAATGGGTACCAACAAGCCAGC
CP-GST-R	TCAGTCAGTCACGATGAATTCCTAGTCATCTGCACCTTCTG

NS3-GST-F	TGATCGAAGGTCGTGGGATCCTAATGAACGTGTTCACATCGTC
NS3-GST-R	TCAGTCAGTCACGATGAATTCCTACAGCACAGCTGGAGAGC
SP-GST-F	TGATCGAAGGTCGTGGGATCCTAATGCAAGACGTACAAAGGAC
SP-GST-R	TCAGTCAGTCACGATGAATTCCTATGTTTTGTGTAGAAGAGG
**Constructs for point mutant**	
$CP^{R77A/T79A/D80A/K87A}$	
$CP^{R77A/T79A/D80A/K87A}$-F	CTGCCTTTGTGAGAGATGTCACTGCGAAAGTGAAAGTGGCGGCTGGAA
$CP^{R77A/T79A/D80A/K87A}$-R	CATCTCTCACAAAGGCAGCGCCTGCCACATATCTCATGGTGATGCACATAGTCAT
$OsOsVIRK1^{K391E}$	
$OsVIRK1^{K391E}$-F	AGAGATTGCAGTGGAGAGAT
$OsVIRK1^{K391E}$-R	GCACTGCAATCTCTTGACCAT
$OsOsVIRK1^{C4A}$	
OsVIRK1-C110A-F	AATGGCACCAGCGCCCGCTCC
OsVIRK1-C110A-R	GCGCTGGTGCCATTGCCGCCG
OsVIRK1-C138A-F	ATCTCCGACTACGCCCTCGTCC
OsVIRK1-C138A-R	GTAGTCGGAGATGATCACGGC
OsVIRK1-C223A-F	GCCGCCGGCGACGCCTACCGC
OsVIRK1-C223A-R	TCGCCGGCGGCGAGGTCGCGG
OsVIRK1-C251A-F	TCACGAGGAGCGCCTCCATCC
OsVIRK1-C251A-R	GCTCCTCGTGATGACCTGCCC
NS3-117D-F	GACCCCCAgatACAAATTGCTGGACATGCAGAA
NS3-117D-R	ATTTGTatcTGGGGGTCTTGGCTTCACTTCAG
NS3-118D-F	CCCCAGATgatAATTGCTGGACATGCAGAATGTC
NS3-118D-R	GCAATTatcATCTGGGGGTCTTGGCTTCACTT
NS3-133D-F	ccctttgacgttcctagtgTCAAGGGATTTCCTCCAGATGC
NS3-133D-R	actaggaacgtcaaagggcAAATTGTCTTTGGACATTCTGCAT
NS3-182D-F	catttctgacgataagccaCCTCTTAGTGCACGTTATGTTAAGTACG
NS3-182D-R	gcttatcgtcagaaatgacatACTTATGCCTTTTCTTACTAGGTGATCT
NS3-188D-F	cacctcttgatgcacgttatgttaagTACGTTGATTCTAGTACACTAGAACCCTC
NS3-188D-R	aacgtgcatcaagaggtggcttATCGGAAGAAATGACATACTTATGCC
**RT-qPCR**	
CP-F	RTTGACAGACATACCAGCCAG
CP-R	CATCATTCACTCCTTCCAAATAACY
OsVIRK1-F	TGGAAGCGAGCGTAGCAAA

OsVIRK1-R	GCCATGTATCCGTAGGTTCCAG
AGO18-F	TGTTCGTCCAGGCACAGTAG
AGO18-R	GCGGTGAAGTTGTTGTCGTC
AOS2-F	GTTGACAACAAGCAGTGCCC
AOS2-R	CGGAGGTTGAAGCTTTGGTG
CM-LOX2-F	GTACGCTGGGTTCACAGCTC
CM-LOX2-R	TCAGATGGATGTGCTGTTGG
**gRNA (Sequence 5’-3’)**	
OsOsVIRK1-1	GGTCGACGACGGTGCCTTCG
OsOsVIRK1-2	GCGCGCTCGGGCTTGACTCG
